# Updated meta-analysis of the role of *APOE* ε2/ε3/ε4 alleles in frontotemporal lobar degeneration

**DOI:** 10.18632/oncotarget.17341

**Published:** 2017-04-21

**Authors:** Wen-Hua Su, Zhi-Hong Shi, Shu-Ling Liu, Xiao-Dan Wang, Shuai Liu, Yong Ji

**Affiliations:** ^1^ Department of Neurology, Tianjin Huanhu Hospital, Tianjin, China; ^2^ Tianjin Key Laboratory of Cerebral Vascular and Neurodegenerative Diseases, Tianjin Huanhu Hospital, Tianjin, China

**Keywords:** APOE, FTLD, allele, genotype, meta-analysis

## Abstract

We performed an updated meta-analysis to assess the role of the ε2/ε3/ε4 alleles of Apolipoprotein E gene (APOE) in frontotemporal lobar degeneration (FTLD). The relevant articles were retrieved from PubMed, CENTRAL, EMBASE and Web of Science databases, and 51 eligible case-control studies with 5123 cases and 20566 controls were selected after screening according to inclusion and exclusion criteria. Our analysis demonstrated that APOE ε4 was associated with increased FTLD risk in all genetic models (ε4 vs. ε3 allele, ε4 vs. ε2 allele, ε4 vs. ε2+ε3+ε4 allele, ε4 vs. ε2+ε3+ε4 carrier, ε4ε4 vs. ε3ε3, ε3ε4 vs. ε3ε3, ε3ε4+ε4ε4 vs. ε3ε3, ε4ε4 vs. ε3ε3+ε3ε4, all *P* < 0.01, odds ratio [OR] > 1). Subgroup analysis revealed significant association between *APOE* ε4 and FTLD (*P* < 0.01, OR > 1) for the Caucasian, Italian, population based (PB), *P* > 0.05 value of the Hardy-Weinberg Equilibrium (HWE), Newcastle-Ottawa scale score > 6, and behavioral variant frontotemporal dementia (bvFTD) subgroups. However, there was no significant association between the APOE ε2 allele and FTLD (*P* > 0.05) in most genetic models and sub-group analyses. Begg's and Egger's tests also revealed no publication bias, and sensitivity analysis showed that our data analysis was robust. Thus our meta-analyses suggest that APOE ε4 is a genetic risk factor in patients with FTLD.

## INTRODUCTION

Frontotemporal lobar degeneration (FTLD) is a common form of dementia that is characterized by focal atrophy of frontal and/or anterior temporal brain lobes [1]. The distinct clinical subtypes of FTLD include behavior variant frontotemporal dementia (bvFTD), semantic dementia (SD) and progressive non-fluent aphasia (PNFA) [2, 3]. Several genetic variants are associated with FTLD [4–6]. In the Italian population, C276T polymorphism of *neuronal nitric oxide synthase* (*nNOS*) gene is linked to increased susceptibility to sporadic FTLD [5]. Conversely, A2518G polymorphism in *monocyte chemotactic protein 1 (MCP-1*) gene is a protective factor of sporadic FTLD [6].

Human Apolipoprotein E (*APOE*) gene that is located on chromosome 19 is involved in lipid homeostasis and is implicated in cardiovascular disease [7, 8]. Altered structure and function of ApoE protein is associated with neurodegenerative disorders such as Alzheimer's disease (AD) [8]. *APOE* gene has three common alleles (ε2, ε3 and ε4) and six related genotypes (ε3ε3, ε3ε2, ε2ε2, ε3ε4, ε4ε4, and ε2ε4) and distinct pathological roles have been attributed to all 3 alleles of *APOE*, namely, ε2, ε3, and ε4 [8]. The conclusions of various studies that have investigated the role of *APOE* polymorphism in FTLD have been inconsistent and contradictory. For instance, *APOE* ε4 was associated with increased FTLD risk in the Dutch population [9]. However, a negative association was reported between *APOE* polymorphism and FTLD risk in German patients [10]. In addition, genome wide association studies (GWAS) data of FTLD did not confirm a positive association with the *APOE* gene [11, 12].

So far, only two meta-analyses have reported on the relationship between *APOE* polymorphism and susceptibility to FTLD [13, 14]. Since many new studies have published on since 2013, we conducted an updated meta-analysis to reassess this association by systematically retrieving, screening and enrolling the available case-control studies to determine the association between *APOE* polymorphism and FTLD risk.

## RESULTS

### Selection criteria for eligible studies in the meta-analysis

**Figure 1:**
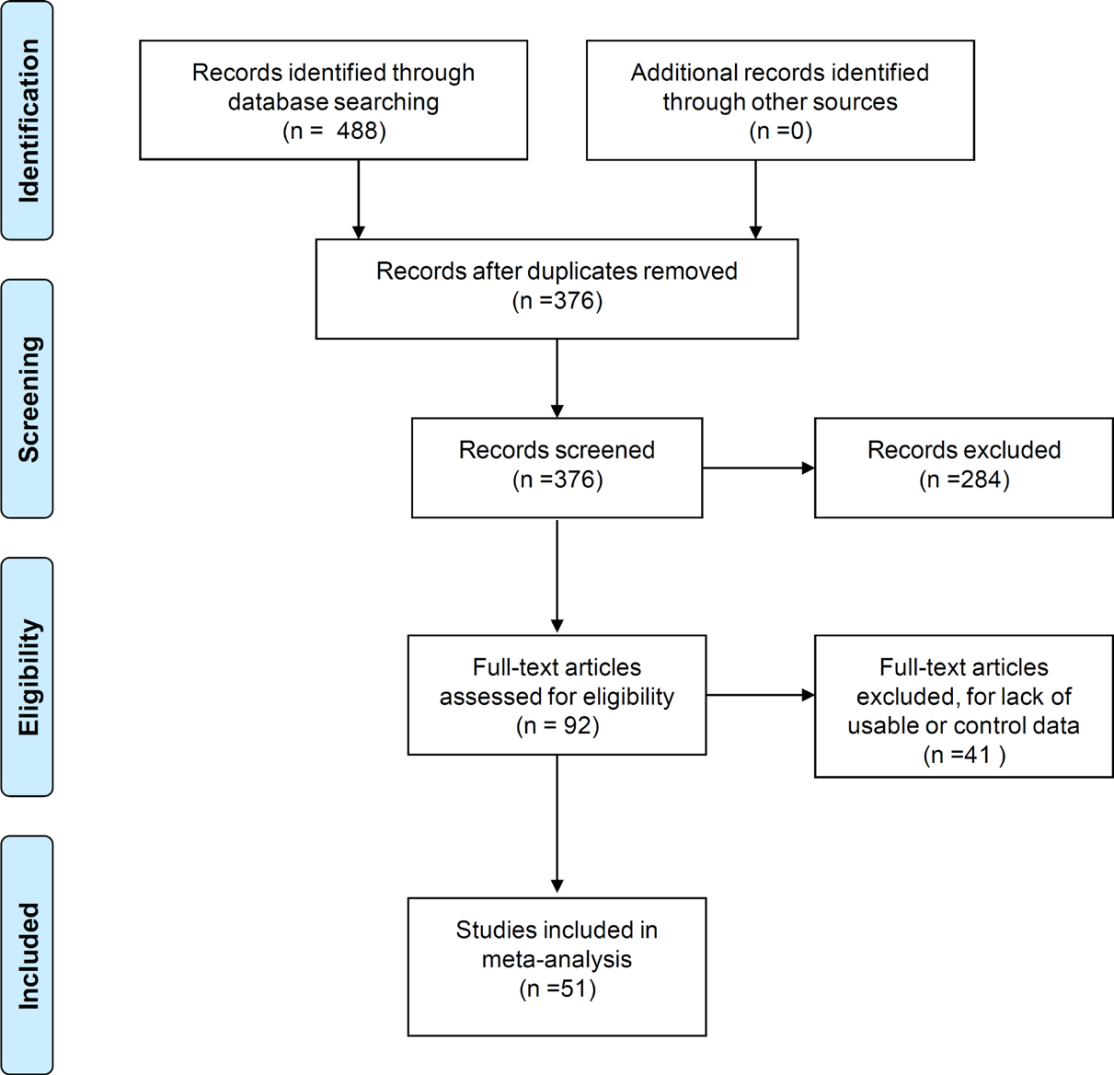
Flow diagram of database search and study selection

Figure 1 shows the flow diagram of methodology used to search databases and select relevant studies based on “Preferred Reporting Items for Systematic Reviews and Meta-Analyses” (PRISMA). A total of 488 records were initially identified by searching four online databases, namely PubMed (*n* = 74), Cochrane Central Register of Controlled Trials (CENTRAL, *n* = 0), Excerpta Medica Database (EMBASE, *n* = 290) and Web of Science (WOS, *n* = 124). We removed 112 duplicate records after identifying them on Endnote. Further, 284 records that included case reports, posters, book articles, reviews, meeting abstracts (*n* = 53), non-FTLD, non-*ApoE*, non-clinical, non-mutation data (*n* = 223), and meta-analysis (*n* = 8) were also excluded. The remaining 92 full-text articles were then assessed for eligibility that resulted in excluding 41 articles for lack of relevant or control data. Finally, 51 case-control studies with 5123 cases and 20566 controls were included in our meta-analysis [5, 6, 9, 10, 13, 15–60]. The NOS assessment showed that three studies had a NOS score of 5 [39, 46, 47] and another three studies had a NOS score of 6 [26, 28, 32] indicating the medium-quality. The other 45 studies [5, 6, 9, 10, 13, 15–25, 27, 29–31, 33–38, 40–45, 48–60] were of high-quality with NOS scores > 6. Supplementary Table 1 shows the characteristics of eligible studies.

### *APOE* polymorphism and FTLD risk meta-analysis

The pooled values of OR and 95% confidence interval (CI) were analyzed by Mantel-Haenszel statistics to identify associations between *APOE* ε2, ε3, ε4 alleles and FTLD risks. As shown in Table 1, increased FTLD risk was observed in ε4 vs. ε3 allele model (*P* < 0.001, OR = 1.66, 95% CI = 1.35–2.03), ε4 vs. ε2 allele model (*P* = 0.008, OR = 1.52, 95% CI = 1.12–2.06), ε4 vs. ε2+ε3+ε4 allele model (*P* < 0.001, OR = 1.52, 95% CI = 1.31–1.76), ε4 vs. ε2+ε3+ε4 carrier model (*P* < 0.001, OR = 1.50, 95% CI = 1.32–1.70). Similarly, increased risk was observed for the genetic models of ε4ε4 vs. ε3ε3 (*P* < 0.001, OR = 3.23, 95% CI = 2.27–4.60), ε3ε4 vs. ε3ε3 (*P* < 0.001, OR = 1.62, 95% CI = 1.25–2.10), ε3ε4+ε4ε4 vs. ε3ε3 (*P* < 0.001, OR = 1.70, 95% CI = 1.33–2.19), and ε4ε4 vs. ε3ε3+ε3ε4 (*P* < 0.001, OR = 2.82, 95% CI = 1.99–3.98) as shown in Table 1. These data demonstrated that the *APOE* ε4 allele increased FTLD susceptibility in a dose-dependent manner.

**Table 1:** Meta-analysis for the association between *APOE* polymorphism and FTLD risks

Comparison	Study number	Sample size (case/control)	Association Test		Heterogeneity		Model
			OR (95% CI)	*P*	*I*^2^	*P*	
**ε4 vs. ε3 allele**	34	2072/13661	1.66 (1.35–2.03)	**< 0.001**	68.7%	< 0.001	Random
**ε4 vs. ε2 allele**	34	2072/13661	1.52 (1.12–2.06)	**0.008**	60.8%	< 0.001	Random
**ε4 vs. ε2+ε3+ε4 allele**	40	2417/15059	1.52 (1.31–1.76)	**< 0.001**	51.3%	< 0.001	Random
**ε4 vs. ε2+ε3+ε4 carrier**	47	3511/18046	1.50 (1.32–1.70)	**< 0.001**	40.9%	0.002	Random
**ε4ε4 vs. ε3ε3**	30	1650/11634	3.23 (2.27–4.60)	**< 0.001**	0.0%	0.922	Fixed
**ε3ε4 vs. ε3ε3**	32	1696/11700	1.62 (1.25–2.10)	**< 0.001**	67.3%	< 0.001	Random
**ε3ε4+ε4ε4 vs. ε3ε3**	32	1696/11700	1.70 (1.33–2.19)	**< 0.001**	67.6%	< 0.001	Random
**ε4ε4 vs. ε3ε3+ε3ε4**	30	1650/11634	2.82 (1.99–3.98)	**< 0.001**	0.0%	0.962	Fixed
**ε2 vs. ε3 allele**	34	2072/13661	1.09 (0.87–1,37)	0.462	51.5%	< 0.001	Random
**ε2 vs. ε2+ε3+ε4 allele**	34	2072/13661	1.01 (0.82–1.24)	0.953	43.1%	0.005	Random
**ε2 vs. ε2+ε3+ε4 carrier**	32	1936/13591	0.93 (0.74–1.17)	0.545	42.3%	0.007	Random
**ε2ε2 vs. ε3ε3**	22	944/9708	1.74 (1.03–2.96)	**0.039**	0.0%	0.774	Fixed
**ε3ε2 vs. ε3ε3**	32	1346/10740	0.87 (0.73–1.04)	0.132	24.2%	0.110	Fixed
**ε3ε2+ε2ε2 vs. ε3ε3**	32	1346/10740	0.95 (0.72–1.23)	0.678	41.6%	0.008	Random
**ε2ε2 vs. ε3ε3+ε3ε2**	22	944/9708	1.84 (1.08–3.12)	**0.024**	0.0%	0.842	Fixed

*P* < 0.05 of association test is shown in bold.

**Figure 2:**
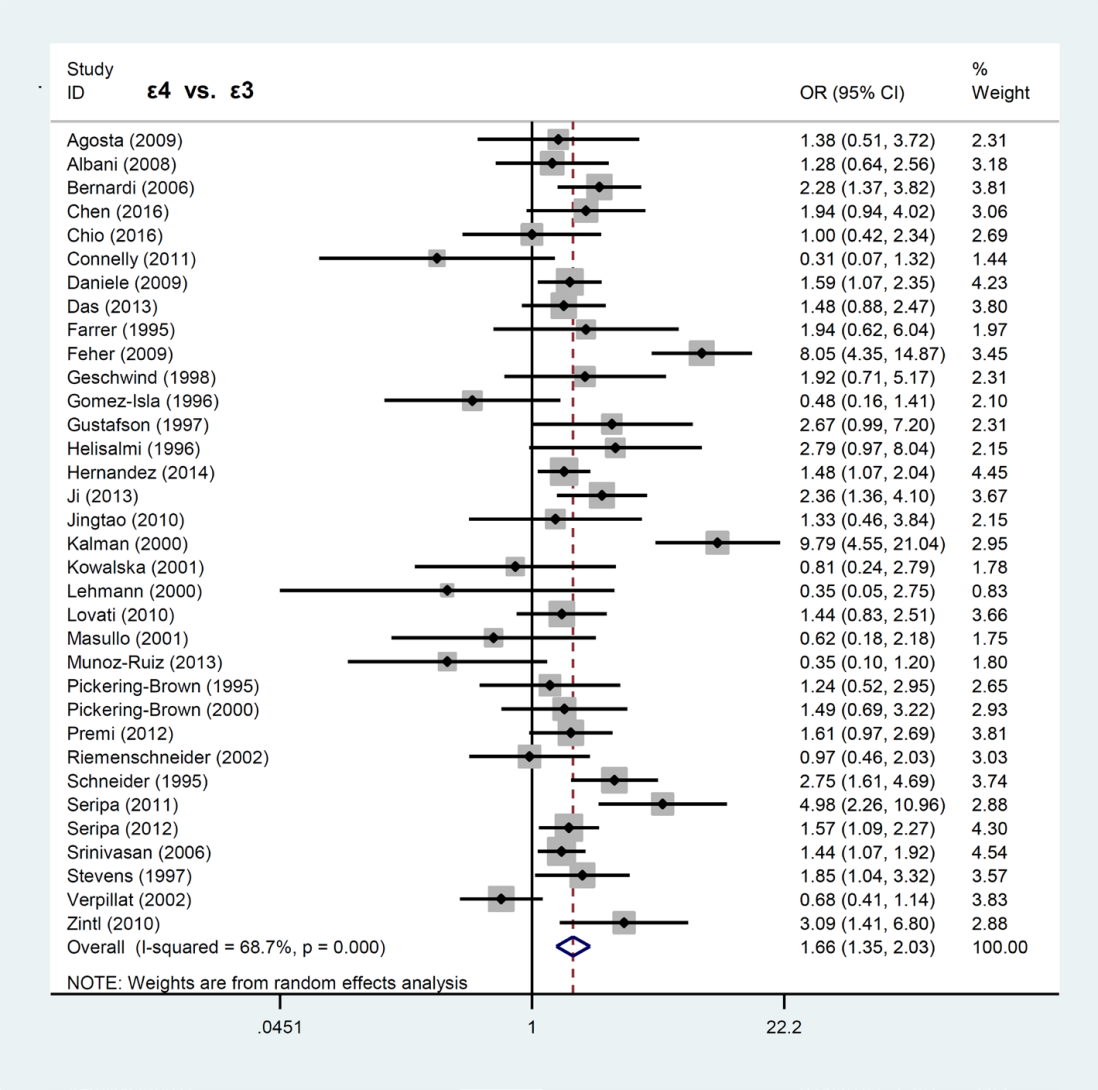
Forest plot of meta-analysis of the ε4 vs. ε3 allele model

**Figure 3:**
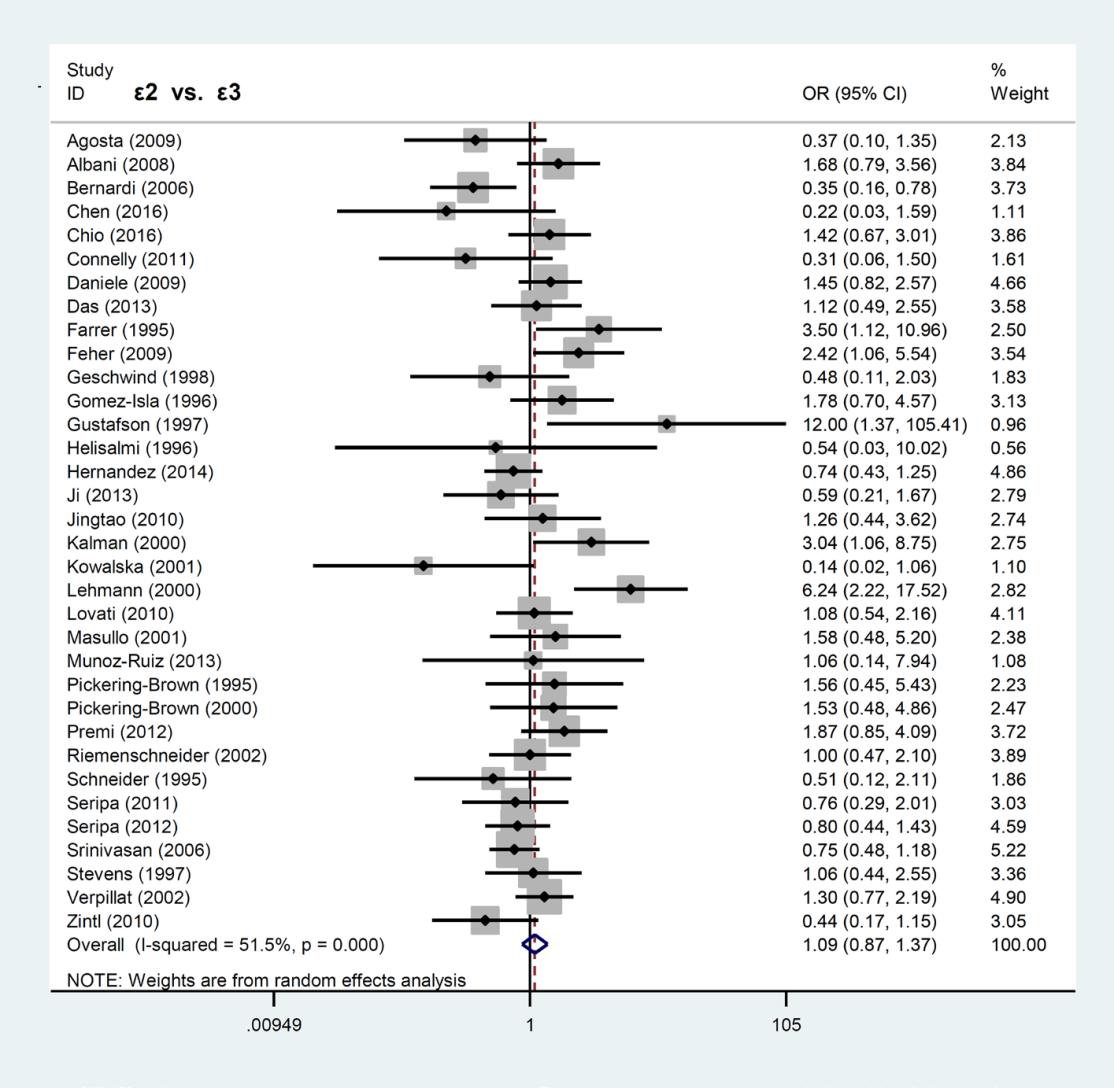
Forest plot of meta-analysis of the ε2 vs. ε3 allele model

In contrast, *APOE* ε2 allele was not associated with FTLD risk. Our analyses for *APOE* ε2 showed significant difference only in the models of ε2ε2 vs. ε3ε3 (*P* = 0.039, OR = 1.74, 95% CI = 1.03–2.96) and ε2ε2 vs. ε3ε3+ε3ε2 (*P* = 0.024, OR = 1.84, 95% CI = 1.08–3.12), but not others (all *P* > 0.05). The forest plots for the allele models of ε4 vs. ε3 and ε2 vs. ε3 are shown in Figures 2 and 3, respectively.

### Subgroup analysis of *APOE* polymorphism and FTLD risk

Next, we performed a series of subgroup analyses based on ethnicity (Caucasian and Asian), country (Italy, China, USA and UK), source of control (PB and HB), clinical subtypes (bvFTD, SD, PNFA, FTLD MND-, FTLD MND+), HWE (*P* value of HWE > 0.05 and < 0.05), and NOS (score > 6 and < = 6). We observed that Caucasian, Italian, PB, *P* value of HWE > 0.05, and NOS score > 6 subgroups for *APOE* ε4 demonstrated increased FTLD risk in the following models: ε4 vs. ε3 (Table 2, all *P* < 0.01, OR > 1); ε4 vs. ε2 (Table 2, all *P* < 0.05, OR > 1); ε4 vs. ε2+ε3+ε4 allele (Table 2, all *P* < 0.001, OR > 1); ε4ε4 vs. ε3ε3 (Table 3, all *P* < 0.001, OR > 1); ε3ε4 vs. ε3ε3 (Table 3, all *P* < 0.01, OR > 1); ε3ε4+ε4ε4 vs. ε3ε3 (Table 4, all *P* < 0.01, OR > 1); and ε4ε4 vs. ε3ε3+ε3ε4 (Table 4, all P < 0.01, OR > 1). These data demonstrated that both ε4ε4 and ε3ε4 genotypes of *APOE* conferred increased susceptibility to FTLD in the Caucasian population, especially people of Italian origin.

**Table 2:** Subgroup analysis of association between *APOE* ε4 and FTLD risks for ε4 vs. ε3, ε4 vs. ε2, and ε4 vs. ε2+ε3+ε4 allele models

	ε4 vs. ε3				ε4 vs. ε2				ε4 vs. ε2+ε3+ε4			
**Subgroup**	**Study number**	**Sample size** **(case/control)**	**OR (95 % CI)**	***P***	**Study number**	**Sample size** **(case/control)**	**OR (95 % CI)**	***P***	**Study number**	**Sample size** **(case/control)**	**OR** **(95 % CI)**	***P***
**Ethnicity**												
Caucasian	29	1854/11162	1.66 (1.31–2.09)	**< 0.001**	29	1854/11162	1.41 (1.01–1.97)	**0.043**	35	2199/12560	1.50 (1.28–1.77)	**< 0.001**
Asian	5	218/2499	1.72 (1.26–2.34)	**0.001**	5	218/2499	2.40 (1.12–5.11)	**0.024**	5	218/2499	1.65 (1.22–2.24)	**0.001**
**Country**												
Italy	10	839/2168	1.64 (1.30–2.07)	**< 0.001**	10	839/2168	1.57(0.93–2.65)	0.091	11	848/2193	1.55 (1.26–1.90)	**< 0.001**
China	3	113/2030	2.04 (1.36–3.07)	**0.001**	3	13/2030	2.99 (0.97–9.21)	0.056	3	113/2030	1.94 (1.30–2.90)	**0.001**
USA	4	106/3394	1.60 (0.75–3.40)	0.224	4	106/3394	1.29 (0.29–5.08)	0.733	5	169/3732	1.62 (1.06–2.49)	**0.026**
UK	4	345/962	1.39 (1.08–1.80)	**0.012**	4	345/962	0.74 (0.23–2.38)	0.609	4	345/962	1.32 (1.03–1.70)	**0.028**
**Source of control**												
PB	31	1912/13391	1.70 (1.36–2.11)	**< 0.001**	31	1912/13391	1.53 (1.11–2.12)	**0.009**	37	2257/14789	1.54 (1.32–1.80)	**< 0.001**
HB	3	160/270	1.25 (0.74–2.11)	0.400	3	160/270	1.19 (0.47–3.03)	0.715	3	160/270	1.20 (1.19–1.86)	0.488
**Clinical subtypes**												
bvFTD	4	373/2257	1.57 (1.246–1.99)	**< 0.001**	4	373/2257	2.14 (1.39–3.30)	**0.001**	5	400/2595	1.49 (1.19–1.86)	**< 0.001**
SD	2	59/956	1.09 (0.63–1.90)	0.755	2	59/956	1.31 (0.49–3.47)	0.587	2	59/956	1.09 (0.63–1.89)	0.747
PNFA	1	60/200	1.80 (1.02–3.15)	**0.041**	1	60/200	0.79 (0.30–2.04)	0.620	2	78/538	1.50 (0.91–2.48)	0.116
FTLD MND−	2	50/149	0.68 (0.29–1.59)	0.373	2	50/149	0.36 (0.11–1.17)	0.090	3	123/477	0.81 (0.53–1.23)	0.324
FTLD MND+	3	45/905	1.56 (0.90–2.71)	0.112	2	42/791	2.45 (0.79–7.57)	0.121	4	116/1233	1.30 (0.93–1.83)	0.125
**NOS**												
score > 6	28	1800/11889	1.67 (1.32–2.12)	**< 0.001**	28	1800/11889	1.64 (1.17–2.30)	**0.004**	34	2145/13287	1.54 (1.30–1.82)	**< 0.001**
score < = 6	6	272/1772	1.51 (1.09–2.10)	**0.014**	6	272/1772	0.98 (0.55–1.72)	0.937	6	272/1772	1.36 (0.99–1.88)	0.059

PB: population-based; HB: hospital-based; bvFTD: behavior variant frontotemporal dementia; SD: semantic dementia; PNFA: progressive non-fluent aphasia; FTLD: Frontotemporal lobar degeneration; MND: motor neuron disease; NOS: Newcastle-Ottawa scale; *P <* 0.05 is shown in bold.

**Table 3:** Subgroup analysis of association between *APOE* ε3/ε4 genotype frequency and FTLD risks for ε4ε4 vs. ε3ε3 and ε3ε4 vs. ε3ε3 models

	ε4ε4 vs. ε3ε3				ε3ε4 vs. ε3ε3			
**Subgroup**	**Study number**	**Sample size (case/control)**	**OR (95 % CI)**	***P***	**Study number**	**Sample size (case/control)**	**OR (95 % CI)**	***P***
**Ethnicity**								
Caucasian	25	**1447/9494**	3.34 (2.31–4.83)	**< 0.001**	27	**1493/9560**	1.61 (1.19–2.16)	**0.002**
Asian	5	**203/2140**	2.20 (0.66–7.36)	0.199	5	**203/2140**	1.84 (1.29–2.63)	**0.001**
**Country**								
Italy	9	710/1850	3.71 (1.83–7.51)	**< 0.001**	10	738/1893	1.61 (1.28–2.03)	**< 0.001**
China	3	105/1735	4.36 (0.90–21.21)	0.068	3	105/1735	2.20 (1.37–3.51)	**0.001**
USA	4	91/2905	1.67 (0.42–6.64)	0.464	4	91/2905	1.58 (0.37–6.74)	0.535
UK	2	179/750	3.75 (1.65–8.54)	**0.002**	2	179/750	1.16 (0.66–2.02)	0.606
**Source of control**								
PB	29	1627/11474	3.28 (2.30–4.67)	**< 0.001**	31	1673/11540	1.65 (1.27–2.15)	**< 0.001**
HB	1	23/160	1.27 (0.06–27.36)	0.879	1	23/160	0.59 (0.13–2.69)	0.494
**HWE**								
*P* > 0.05	25	1481/10080	2.92 (1.99–4.30)	**< 0.001**	27	1527/10146	1.55 (1.20–2.01)	**0.001**
*P* < 0.05	5	169/1554	5.58 (2.31–13.47)	**< 0.001**	5	169/1554	1.95 (0.51–7.53)	0.332
**Clinical subtypes**								
bvFTD	3	310/1859	4.42 (1.93–10.09)	**< 0.001**	4	338/1902	1.48 (1.11–1.98)	**0.008**
SD	2	53/816	3.39 (0.82–13.91)	0.091	2	53/816	0.94 (0.46–1.92)	0.866
PNFA	1	56/185	1.28 (0.05–32.17)	0.879	1	56/185	1.85 (0.96–3.58)	0.066
FTLD MND−	1	19/103	1.61 (0.06–41.17)	0.774	1	19/103	0.55 (0.12–2.59)	0.449
FTLD MND+	2	30/734	3.04 (0.53–17.44)	0.212	2	30/734	1.50 (0.69–3.29)	0.306
**NOS**								
score > 6	26	1529/10171	3.32 (2.28–4.82)	**< 0.001**	28	1575/10237	1.67 (1.27–2.21)	**< 0.001**
Score < = 6	4	121/1463	2.58 (0.88–7.59)	0.084	4	121/1463	1.38 (0.82–2.32)	0.222

PB: population-based; HB: hospital-based; HWE: Hardy-Weinberg Equilibrium; bvFTD: behavior variant frontotemporal dementia; SD: semantic dementia; PNFA: progressive non-fluent aphasia; FTLD: Frontotemporal lobar degeneration; MND: motor neuron disease; NOS: Newcastle-Ottawa scale; *P <* 0.05 is shown in bold.

**Table 4:** Subgroup analysis of association between *APOE* ε3/ε4 genotype frequency and FTLD risks for ε3ε4+ε4ε4 vs. ε3ε3 and ε4ε4 vs. ε3ε3+ε3ε4 models

	ε3ε4+ε4ε4 vs. ε3ε3				ε4ε4 vs. ε3ε3+ε3ε4			
**Subgroup**	**Study number**	**Sample size (case/control)**	**OR (95 % CI)**	***P***	**Study number**	**Sample size (case/control)**	**OR (95 % CI)**	***P***
**Ethnicity**								
Caucasian	27	**1493/9560**	1.71 (1.28–2.27)	**< 0.001**	25	1447/9494	2.90 (2.02–4.17)	**< 0.001**
Asian	5	**203/2140**	1.82 (1.26–2.63)	**0.001**	5	203/2140	2.02 (0.61–6.72)	0.252
**Country**								
Italy	10	738/1893	1.67 (1.30–2.16)	**< 0.001**	9	710/1850	3.31 (1.63–6.72)	**0.001**
China	3	105/1735	2.21 (1.40–3.51)	**0.001**	3	105/1735	3.74 (0.77–18.13)	0.101
USA	4	91/2905	1.57 (0.43–5.77)	0.498	4	91/2905	1.21 (0.32–4.57)	0.774
UK	2	179/750	1.14 (0.40–3.27)	0.808	2	179/750	3.57 (1.58–8.08)	**0.002**
**Source of control**								
PB	31	1673/11540	1.74 (1.35–2.24)	**< 0.001**	29	1627/11474	2.85 (2.01–4.04)	**< 0.001**
HB	1	23/160	0.54 (0.12–2.45)	0.425	1	23/160	1.35 (0.06–28.97)	0.848
HWE								
*P* > 0.05	27	1527/10146	1.60 (1.24–2.06)	**< 0.001**	25	1481/10080	2.59 (1.77–3.79)	**< 0.001**
*P* < 0.05	5	169/1554	2.57 (0.88–7.51)	0.085	5	169/1554	4.38 (1.88–10.20)	**0.001**
**Clinical subtypes**								
bvFTD	4	338/1902	1.62 (1.22–2.14)	**0.001**	3	310/1859	3.96 (1.76–8.94)	**0.001**
SD	2	53/816	1.03 (0.54–1.95)	0.935	2	53/816	3.60 (0.88–14.71)	0.074
PNFA	1	56/185	1.80 (0.94–3.47)	0.077	1	56/185	1.09 (0.04–27.09)	0.959
FTLD MND−	1	19/103	0.52 (0.11–2.44)	0.408	1	19/103	1.75 (0.07–44.61)	0.734
FTLD MND+	2	30/734	1.54 (0.72–3.31)	0.263	2	30/734	2.71 (0.48–15.17)	0.257
**NOS**								
score > 6	28	1575/10237	1.75 (1.34–2.29)	**< 0.001**	26	1529/10171	2.86 (1.98–4.12)	**< 0.001**
Score < = 6	4	121/1463	1.43 (0.83–2.46)	**0.203**	4	121/1463	2.51 (0.85–7.40)	0.095

PB: population-based; HB: hospital-based; HWE: Hardy-Weinberg Equilibrium; bvFTD: behavior variant frontotemporal dementia; SD: semantic dementia; PNFA: progressive non-fluent aphasia; FTLD: Frontotemporal lobar degeneration; MND: motor neuron disease; NOS: Newcastle-Ottawa scale; *P* < 0.05 is shown in bold.

Moreover, our analysis for *APOE* ε4 in Asian populations, especially Chinese individuals demonstrated enhanced FTLD risk for the allele (Table 2, ε4 vs. ε3, *P* = 0.001, OR = 2.04; ε4 vs. ε2+ε3+ε4, *P* = 0.001, OR = 1.94), heterozygote (Table 3, ε3ε4 vs. ε3ε3, *P* = 0.001, OR = 2.20), dominant (Table 4, ε3ε4+ε4ε4 vs. ε3ε3, *P* = 0.001, OR = 2.21) and carrier (Supplementary Table 2, ε4 vs. ε2+ε3+ε4 carrier, *P* = 0.003, OR = 1.92) models, but were not significant for homozygote (Table 3, ε4ε4 vs. ε3ε3, *P* = 0.068) and recessive (Table 4, ε4ε4 vs. ε3ε3+ε3ε4, *P* = 0.101) models. These indicated that in the Asian population, including the Chinese individuals, the ε3ε4 genotype was linked to increased FTLD risk. The forest plots of subgroup analysis based on ethnicity for APOE ε4 under all genetic models were shown in Supplementary Figures 1–8.

In addition, stratified analysis of clinical subtypes (bvFTD, SD, PNFA, FTLD with or without motor neuron disease) showed that all genetic models were associated with increased bvFTD risk (Tables 2, 3, 4, Supplementary Table 2, all *P* < 0.01, OR > 1). This suggested that *APOE* ε4 was a risk factor for bvFTD.

In regard to *APOE* ε2, no significant differences were observed in the subgroup analyses for almost all genetic models (Supplementary Tables 2, 3, 4, 5, *P* > 0.05). These findings further confirmed the negative genetic association between *APOE* ε2 and FTLD risks.

### Heterogeneity, publication bias and sensitivity analysis

We assessed heterogeneity between studies by performing the *Q* statistic and *I*^2^ tests. As shown in Table 1, there was no heterogeneity among different studies for the following models: ε4ε4 vs. ε3ε3, ε4ε4 vs. ε3ε3+ε3ε4, ε2ε2 vs. ε3ε3, ε3ε2 vs. ε3ε3, and ε2ε2 vs. ε3ε3+ε3ε2 (all *P* value of heterogeneity > 0.1, *I*^2^< 25 %). Hence, we used the fixed-effect model for their analysis. The random-effect model was applied for others.

**Figure 4:**
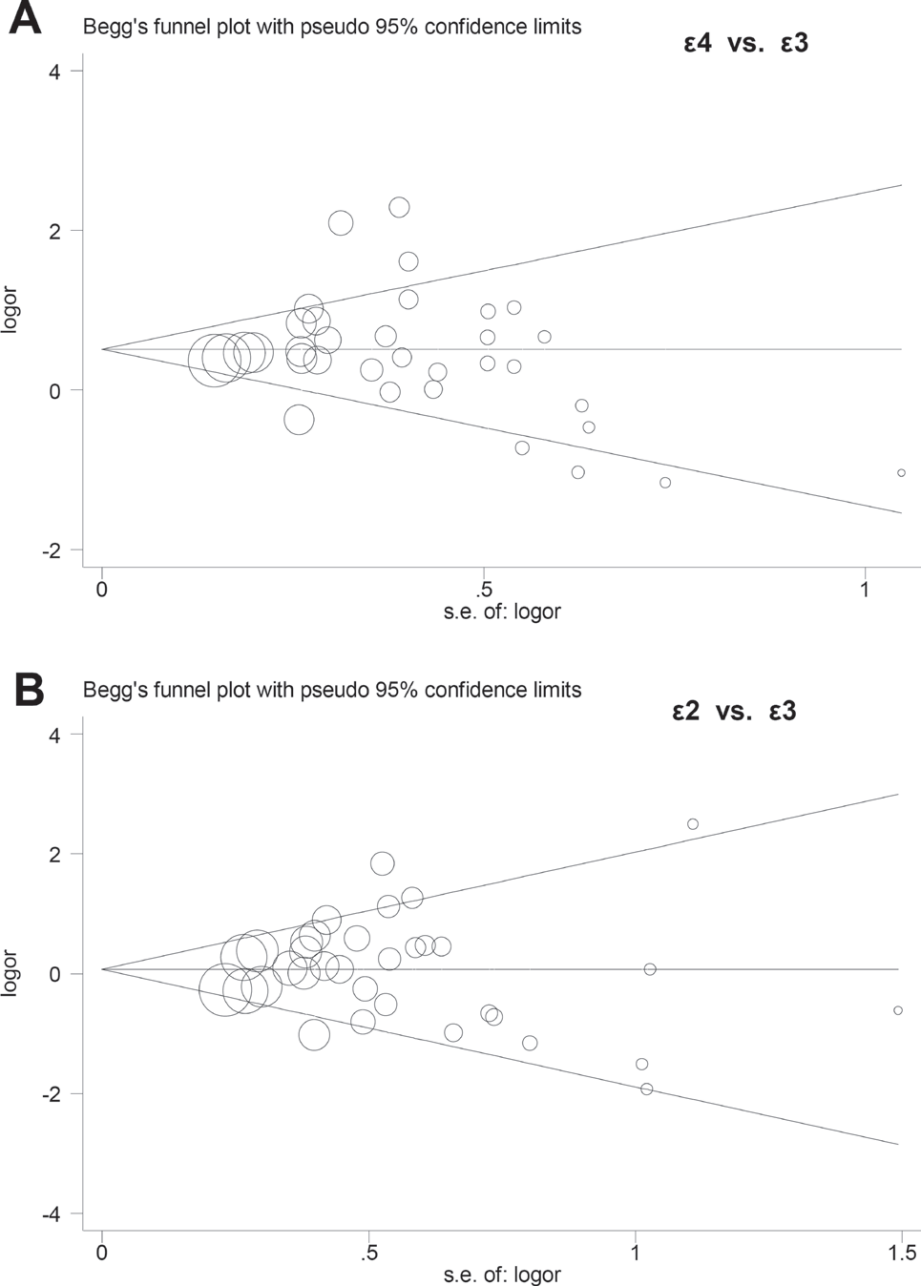
Begg's funnel plots of publication bias (**A**) ε4 vs. ε3 allele model; (**B**) ε2 vs. ε3 allele model.

**Figure 5:**
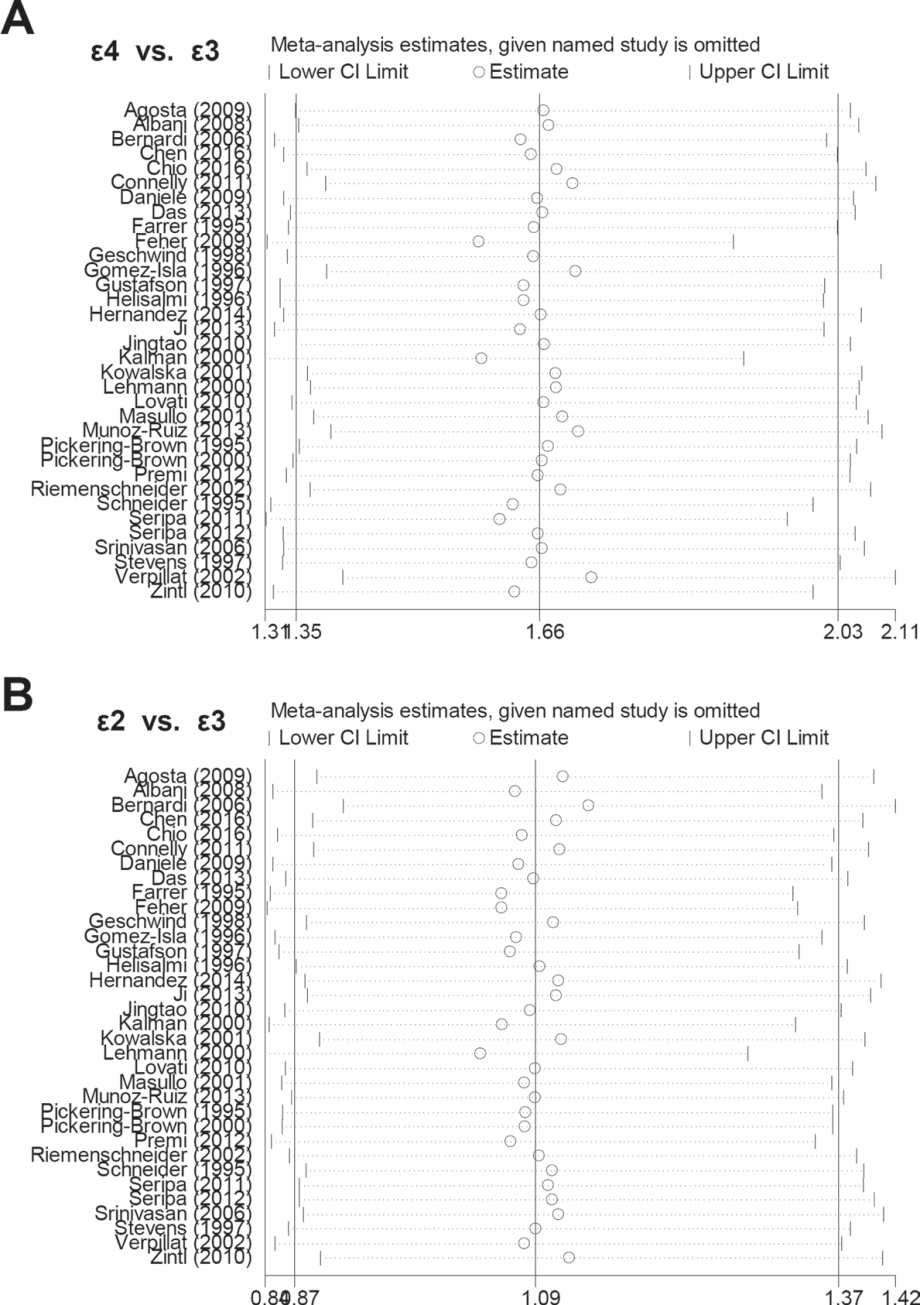
Sensitivity analyses (**A**) ε4 vs. ε3 allele model; (**B**) ε2 vs. ε3 allele model.

In addition, Begg's test and Egger's test analyses suggested absence of publication bias (Supplementary Table 6, all *P* value > 0.1). Begg's funnel plot of publication bias for ε4 vs. ε3 and ε2 vs. ε3 allele models are shown in Figure 4. Furthermore, sensitivity analysis was performed to evaluate the reliability of data and strengthen the validity of genetic relationship. We observed that similar pooled ORs were obtained when individual studies were omitted one by one, thereby indicating that the original statistical data were genuine and robust (Figure 5).

## DISCUSSION

In 2002, Verpillat *et al*. [13] carried out a meta-analysis of 11 studies, and reported that *APOE* ε2 was associated with an increased risk of FTLD in the Caucasian population. However, in 2013, another meta-analysis based on 28 studies by Rubino *et al*. [14] in 2013 showed that FTLD susceptibility was associated with *APOE* ε4, but not ε2. These contradictory conclusions may have been a result of small and different sample sizes.

Recently, mutations in valosin-containing protein (*VCP*), progranulin (GRN), and the microtubule-associated protein tau (*MAPT*) genes were reported by us in 38 Chinese FTLD cases [61]. Further, our analysis of 62 Chinese FTLD patients and 381 sex- and age-matched elderly controls demonstrated significant association between FTLD susceptibility and *APOE* ε4, but not ε2 [36]. However, both conclusions were limited by small sample sizes. Therefore, to comprehensively assess the factors that are associated with FTLD, we enrolled 51 case-control studies and conducted an updated meta-analysis that also included subtype analyses of factors such as country, ethnicity, source of controls and clinical subtypes. Our data demonstrated a strong positive association between *APOE* ε4 and FTLD risks in the allele, homozygote, heterozygote, dominant recessive and carrier models. However, no statistically correlation was observed between *APOE* ε2 and FTLD risks, thereby confirming our previous finding [36] and partly agreeing with the results reported by Verpillat *et al*. [13].

FTLD and Alzheimer's disease (AD) are main contributors to dementia [62]. The molecular mechanisms underlying the role of *APOE* ε4 in the pathogenesis of FTLD and AD are unclear. *APOE* ε4 reduced the clearance of beta-amyloid (Aβ) that resulted in enhanced Aβ deposition within the neurons in the AD mouse model [63, 64]. *APOE* ε4 was also associated with Aβ deposition in the brain of a FTLD case [65]. Hence, the link between *APOE* ε4 and Aβ deposition merits further investigation. In addition, *APOE* ε4 enhanced phosphorylation of tau protein in brains of transgenic mice [66]. Since FTLD-tau is a neuropathological subtype of FTLD [4, 67], abnormal Tau phosphorylation may be partly involved in the pathogenesis of FTLD by *APOE* ε4.

There are several limitations in this meta-analysis that need to be highlighted. Firstly, out of 51 case-control studies included in our pooled analysis, 19 studies [5, 6, 17, 18, 20, 21, 27, 35, 43, 44, 46, 47, 49, 50, 54, 55, 57–59] contained only allele or carrier data and did not provide information regarding the specific genotype frequencies of ε3ε4 and ε3ε2 that could have weakened the statistical output. Secondly, genetic heterogeneity existed between studies for majority of comparisons because of hospital based controls, lack of the pathology or autopsy confirmed FTLD diagnoses, clinical complexity, and pathological heterogeneity. Although poor quality studies were excluded based on NOS analysis, six medium quality articles [26, 28, 32, 39, 46, 47] were still included in the analysis. Hence, more high quality studies with large sample sizes are required to avoid false positives. Thirdly, our meta-analysis included only five articles based on Asian populations [22, 26, 36, 37, 39] compared to 46 articles based on Caucasian populations [5, 6, 9, 10, 13, 15–21, 23–25, 27–35, 38, 40–60]. Among these were 15 articles based on Italian populations [5, 6, 15–17, 19, 20, 23, 25, 41, 42, 48, 52, 53, 59]. In addition, only full-text articles in Chinese or English were collected for this meta-analysis. All these factors might lead to selection bias. Fourthly, bvFTD, the most frequent clinical subtype of FTLD is a clinical syndrome characterized by progressive changes of personality, abnormalities of social behavior and cognitive function, and lack of emotional response [4, 68]. Our subgroup analysis of bvFTD contained seven articles [6, 15, 21, 22, 53, 54, 56] that showed significant association with *APOE* ε4. It is probable that *APOE* ε4 may serve as a disease modifier of bvFTD. However, this result needs to be verified since our analysis was based on a small sample size. Similarly, only four articles for PNFA [6, 21, 48, 54] and five articles for SD [6, 21, 22, 48, 56] were available and therefore the role of *APOE* polymorphisms in PNFA and SD could not be determined conclusively. This was true of the subgroup analysis of FTLD with or without MND. Finally, in view of the unclear etiology of FTLD, more factors, including age at onset, male/female, pathological criteria, clinical presentation, living habits, the combination of *APOE* and other related genes (e.g. *VCP*, *GRN*, *MAPT*) should be considered in future meta-analysis. Also, pathogenesis of *APOE* ε4 in the memory function, behavioral symptoms and brain morphological changes in FTLD-spectrum disease should be investigated.

In conclusion, this meta-analysis demonstrated that *APOE* ε4 was a genetic risk factor for FTLD patients in Caucasian and Asian populations, thereby corroborating the role of *APOE* genetic variants in FTLD. Also, our study demonstrated that *APOE* ε2 was not a susceptibility factor for FTLD.

## MATERIALS AND METHODS

### Database search and study selection

We searched four databases, including PubMed, CENTRAL, EMBASE and WOS until February 27th, 2017 with specific search terms listed in Supplementary Table 7 and identified 488 records. After removing the duplicates by endnote software (Thomson Reuters), the remaining 376 records were screened according to our inclusion/exclusion criteria. We excluded the records of case reports, posters, books, reviews, meeting abstracts, meta-analysis, and the articles with non-FTLD, non-*ApoE*, non-clinical, non-mutation data. The remaining 92 full-text articles were then assessed to identify 51 eligible case-control studies while removing articles that lacked control or other usable data for this meta-analysis. The PRISMA was used in this study [69]. The PRISMA 2009 checklist is shown in Supplementary Table 8.

### Quality assessment of eligible studies and data extraction

Three authors independently assessed the methodological quality of the selected case-control studies using the Newcastle-Ottawa Scale (NOS) (http://www.ohri.ca/programs/clinical_epidemiology/oxford.asp) and extracted the relevant data. Studies with a NOS score > 6 were considered high quality, whereas studies with NOS score < 5 were considered poor and removed from the included studies. Whenever there was a disagreement, it was resolved by discussion among the three authors. The following information was collected from all the selected studies and summarized: first author, year of publication, country, ethnicity, genotype distributions (ε3ε3, ε3ε2, ε2ε2, ε3ε4, ε4ε4, and ε2ε4) in case group and control group, clinical subtypes of case, source of control, and genotyping assay. The first or the corresponding author was contacted by email whenever relevant data was not available.

### Statistical analyses

Stata/SE 12.0 software (StataCorp, USA) was used for Mantel-Haenszel statistic, Q statistic and I^2^ tests from *P* values, pooled ORs, and 95% CIs. *P*<0.05 was considered statistically significant. Six genetic models, namely allele (ε4 vs. ε3; ε2 vs. ε3; ε4 vs. ε2; ε4 vs. ε2+ε3+ε4, ε2 vs. ε2+ε3+ε4), homozygote (ε4ε4 vs. ε3ε3, ε2ε2 vs. ε3ε3), heterozygote (ε3ε4 vs. ε3ε3, ε3ε2 vs. ε3ε3), dominant (ε3ε4+ε4ε4 vs. ε3ε3, ε3ε2+ε2ε2 vs. ε3ε3), recessive (ε4ε4 vs. ε3ε3+ε3ε4, ε2ε2 vs. ε3ε3+ε3ε2) or carrier (ε4 vs. ε2+ε3+ε4 carrier; ε2 vs. ε2+ε3+ε4 carrier) were used and Hardy-Weinberg Equilibrium (HWE) was calculated by chi-squared test. *P* values of *Q* statistic >0.1 or *I*^2^ values ≤ 25% indicated heterogeneity between studies and the fixed-effect model was used for analysis. If not, the random-effect model was used. Subgroup analyses were performed based on ethnicity, country, source of control, clinical subtypes, HWE, and NOS score. Furthermore, Begg's funnel plot (Begg's test) and Egger's publication bias plot (Egger's test) was used to evaluate the potential publication bias. The *P* value of Begg's test and Egger's test > 0.05 was regarded as the absence of publication bias. Sensitivity analysis was also performed to evaluate the stability of statistical results.

## SUPPLEMENTARY MATERIALS FIGURES AND TABLES








