# Supplementary material for: Updated meta-analysis of the role of APOE ε2/ε3/ε4 alleles in frontotemporal lobar degeneration
Source: Oncotarget. 2017 Apr 21;8(27):43721–32. doi: 10.18632/oncotarget.17341 (PMC5546436; doi:10.18632/oncotarget.17341)
Supplement: Supplementary file 1 [file oncotarget-08-43721-s001.pdf]

Updated meta-analysis of the role of *APOE* ε2/ε3/ε4 alleles in frontotemporal lobar degeneration

Supplementary Materials

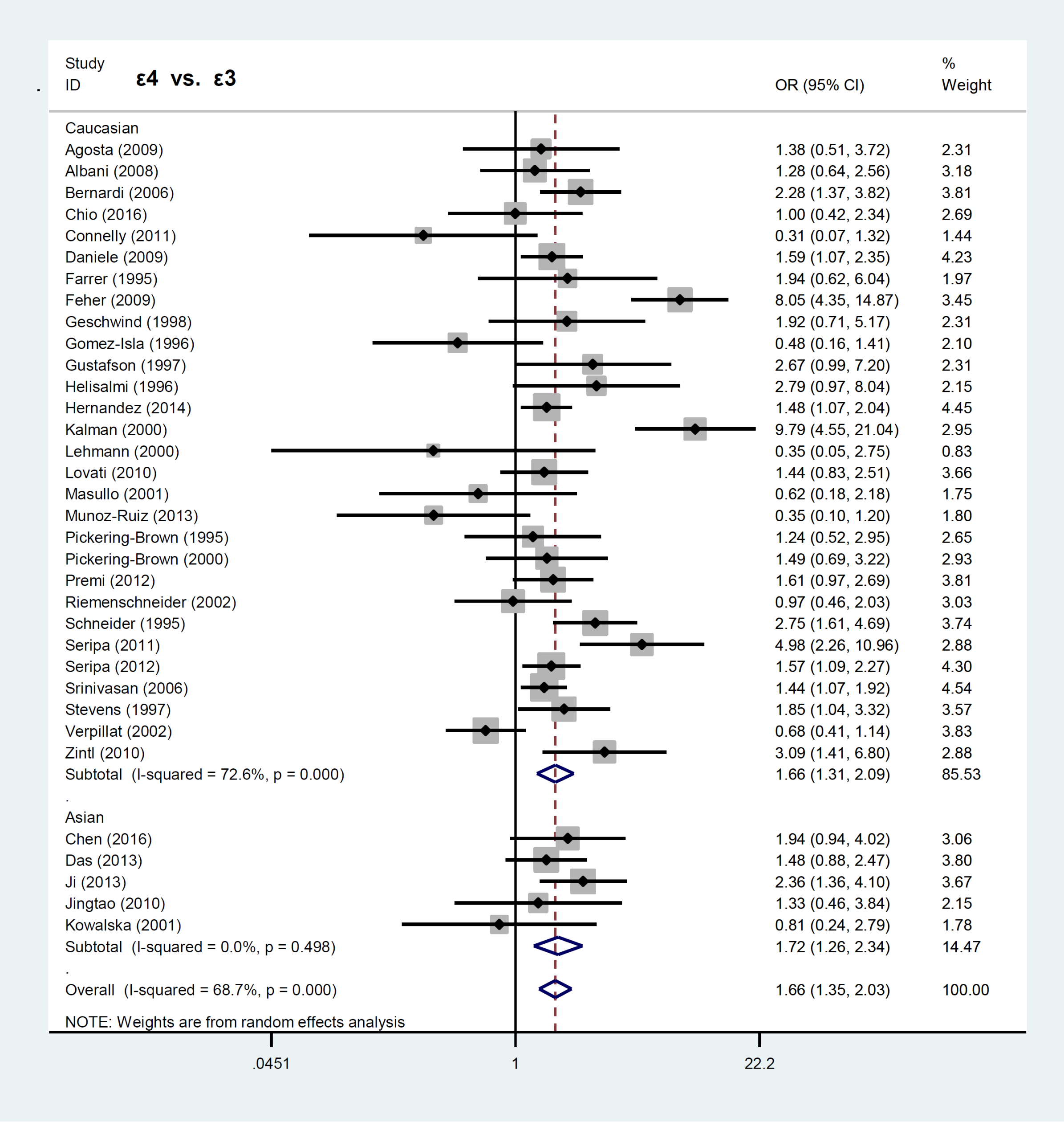

Supplementary Figure 1: Forest plot of subgroup analysis (Asian/Caucasian) of the ε4 vs. ε3 allele model.

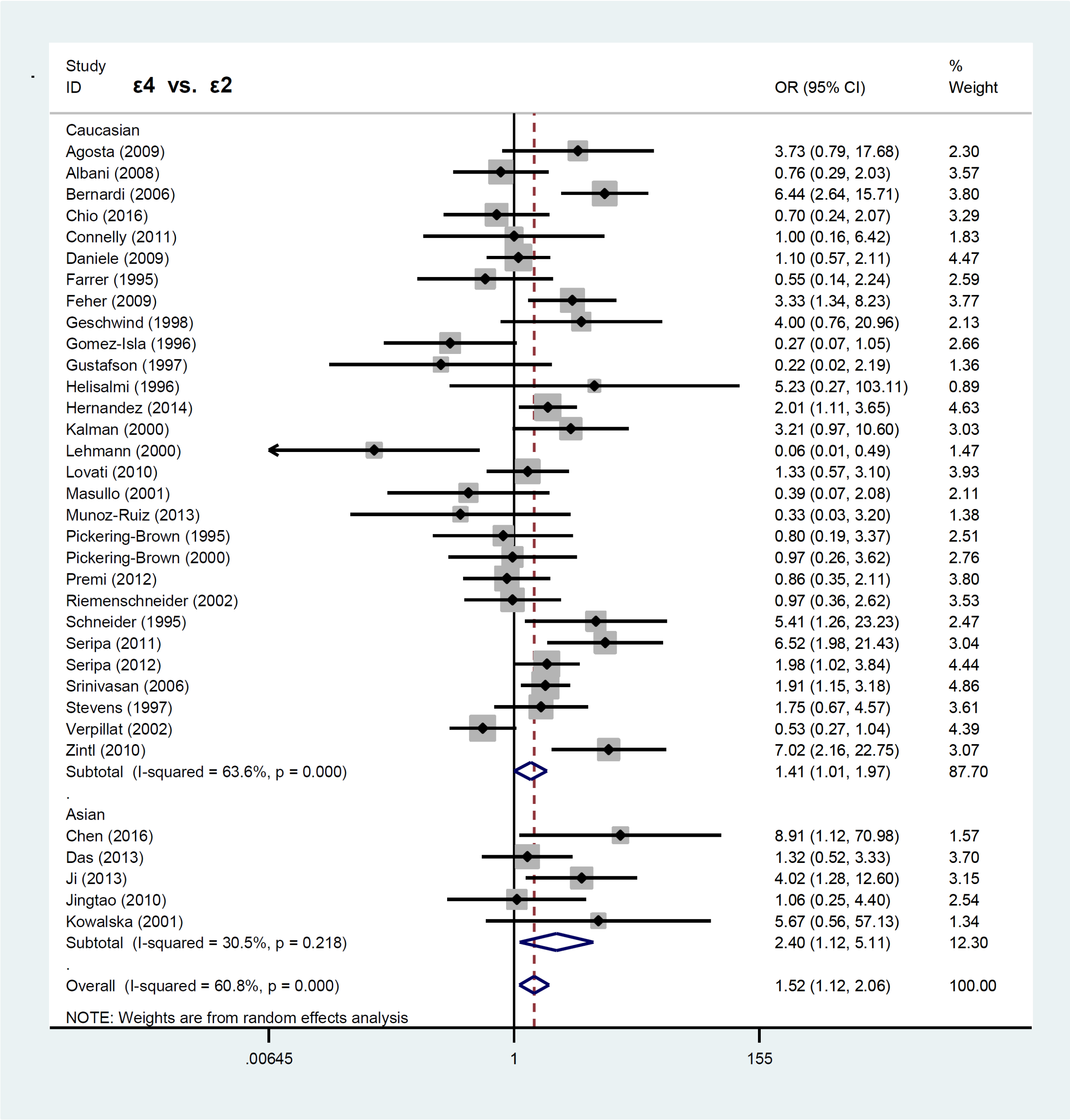

Supplementary Figure 2: Forest plot of subgroup analysis (Asian/Caucasian) of the ε4 vs. ε2 allele model.

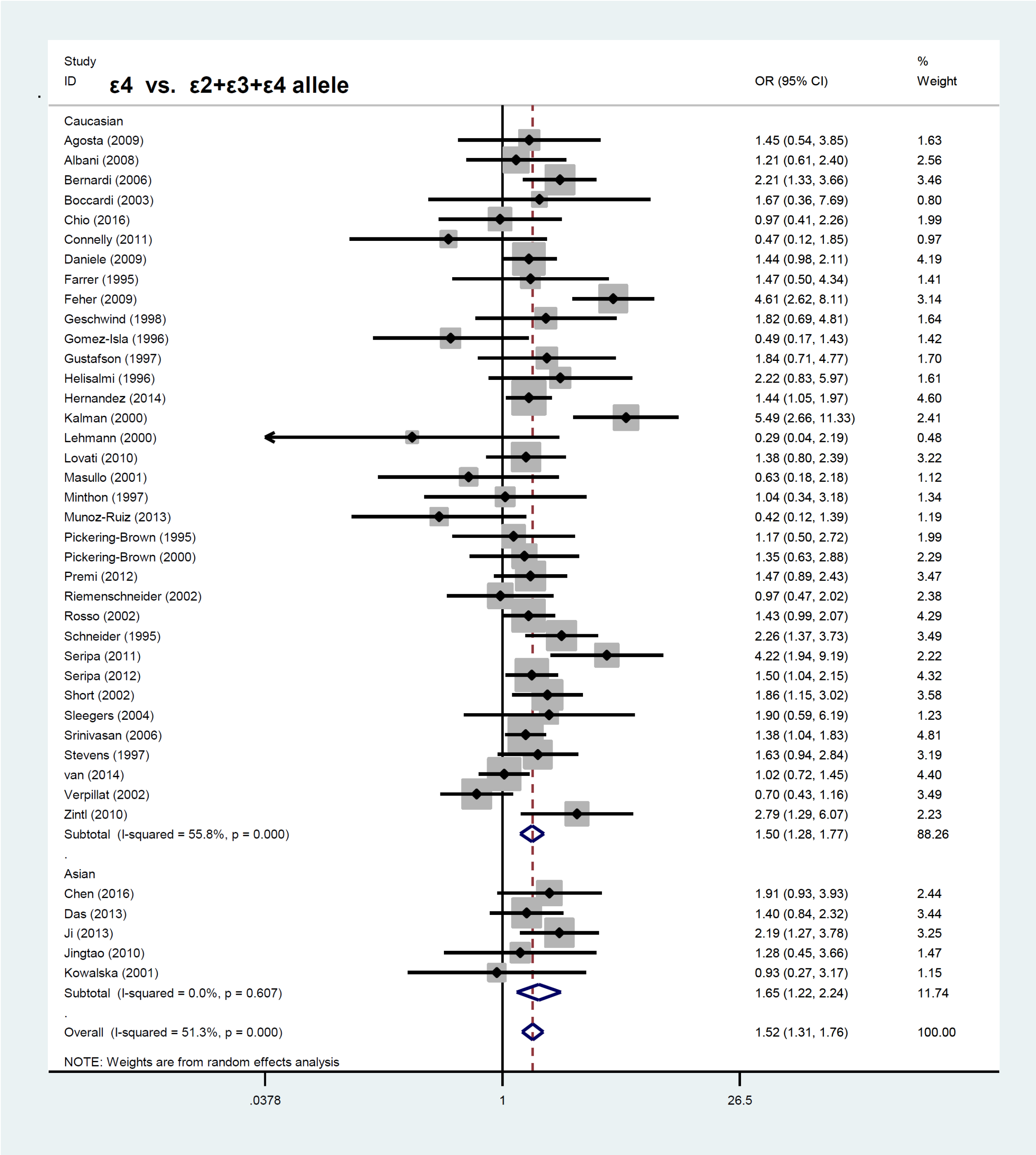

Supplementary Figure 3: Forest plot of subgroup analysis (Asian/Caucasian) of the ε4 vs. ε2+ε3+ε4 allele model.

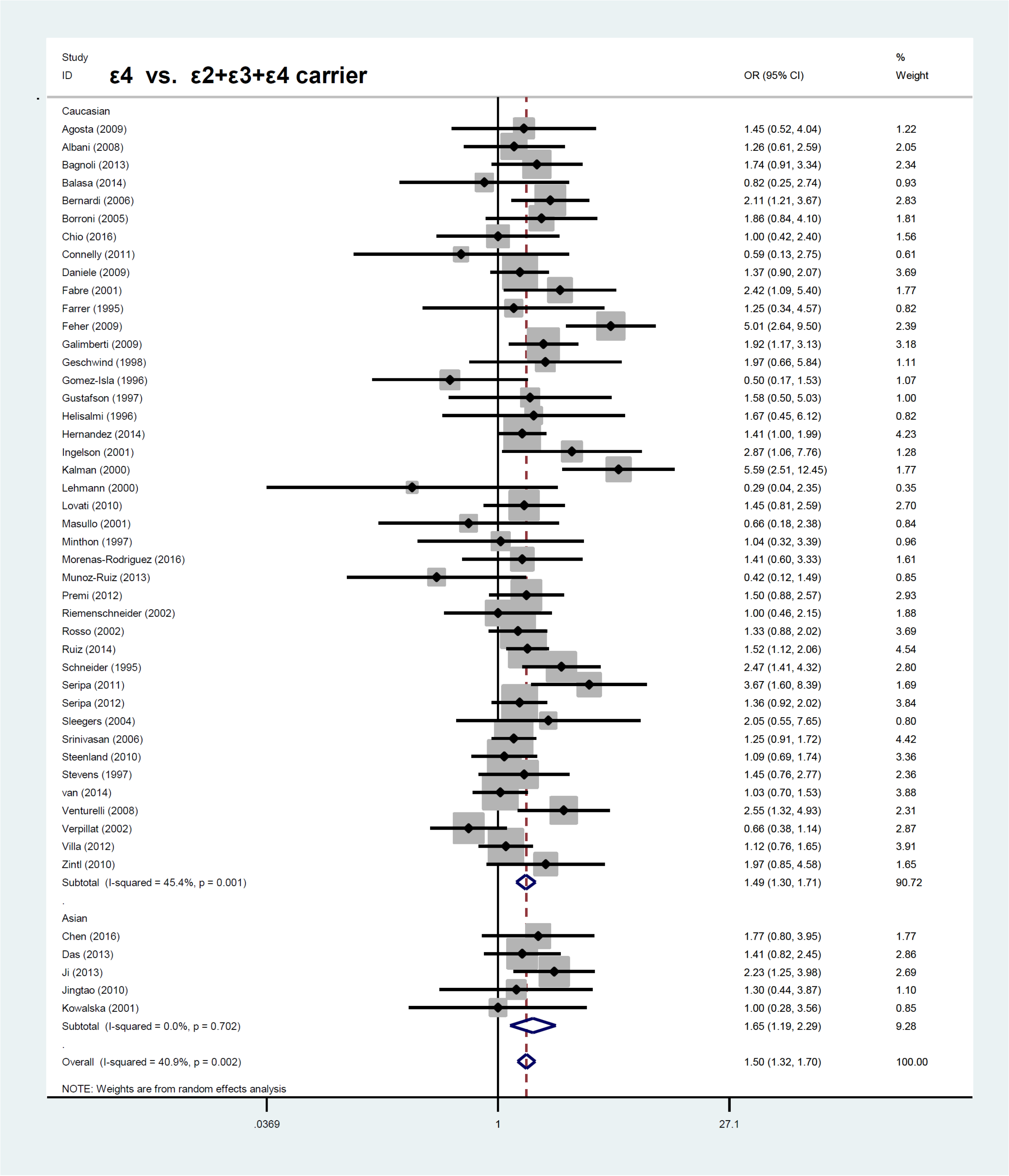

Supplementary Figure 4: Forest plot of subgroup analysis (Asian/Caucasian) of the ε4 vs. ε2+ε3+ε4 carrier model.

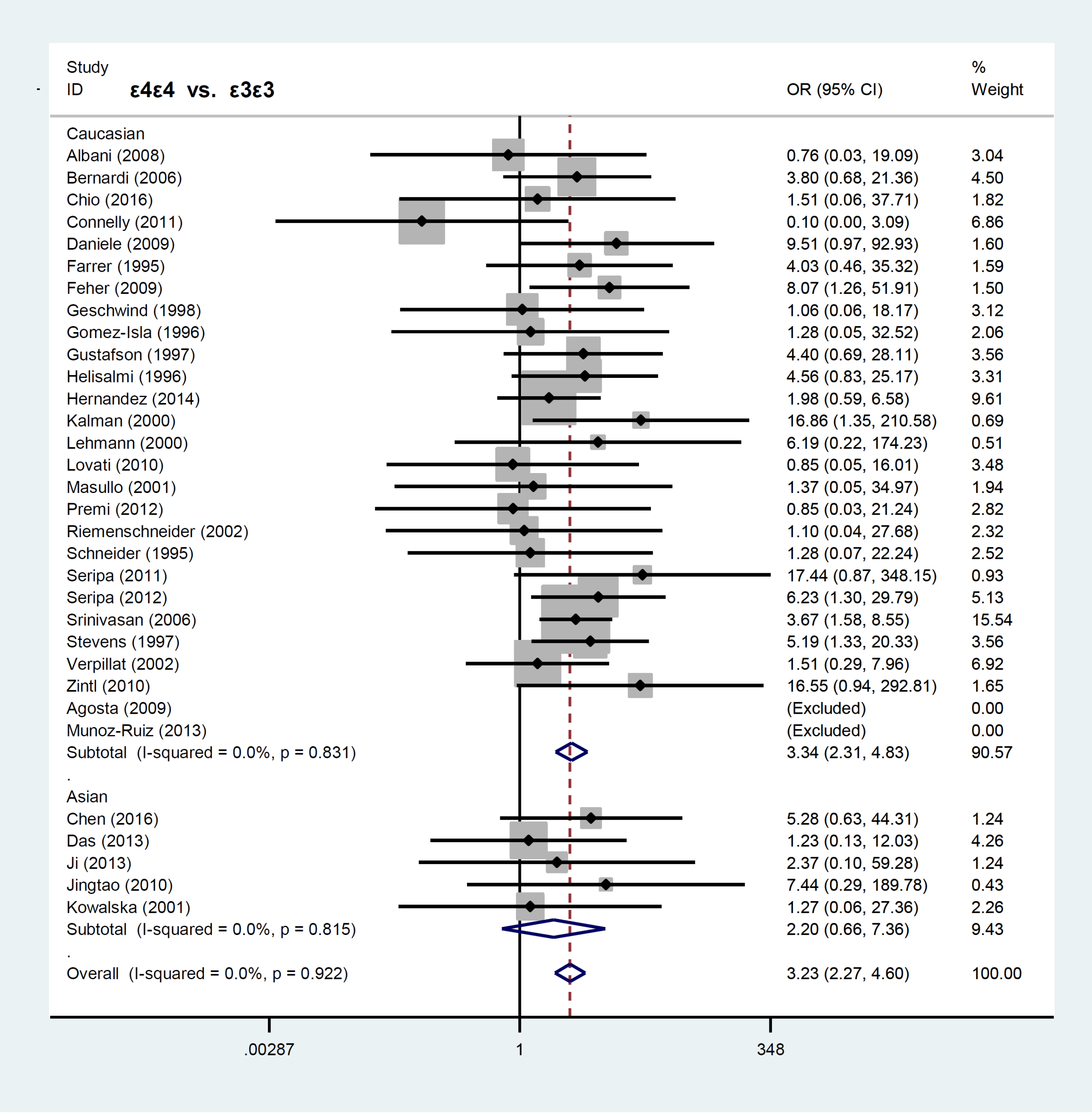

Supplementary Figure 5: Forest plot of subgroup analysis (Asian/Caucasian) of the ε4ε4 vs. ε3ε3 model.

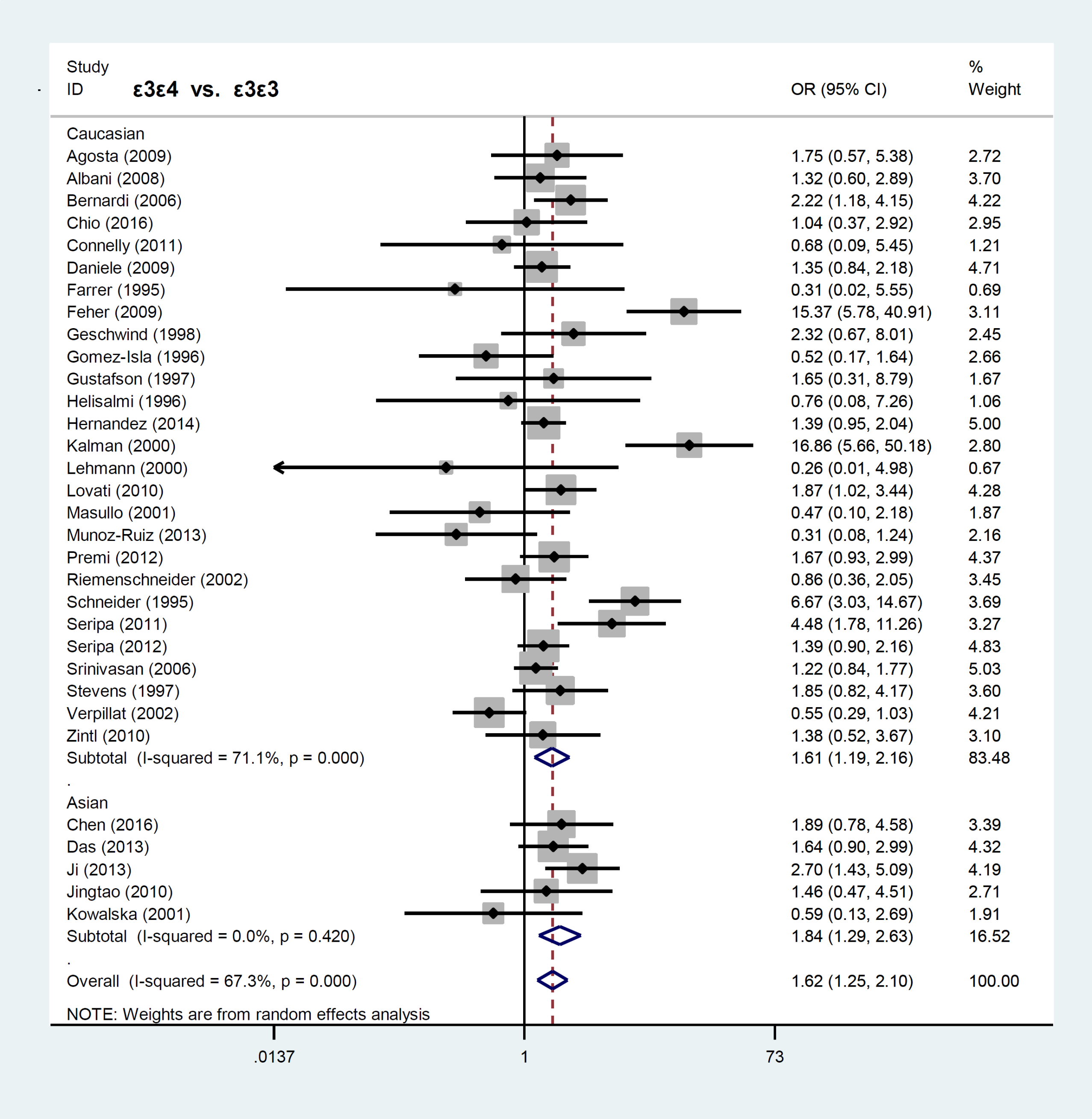

Supplementary Figure 6: Forest plot of subgroup analysis (Asian/Caucasian) of the ε3ε4 vs. ε3ε3 model.

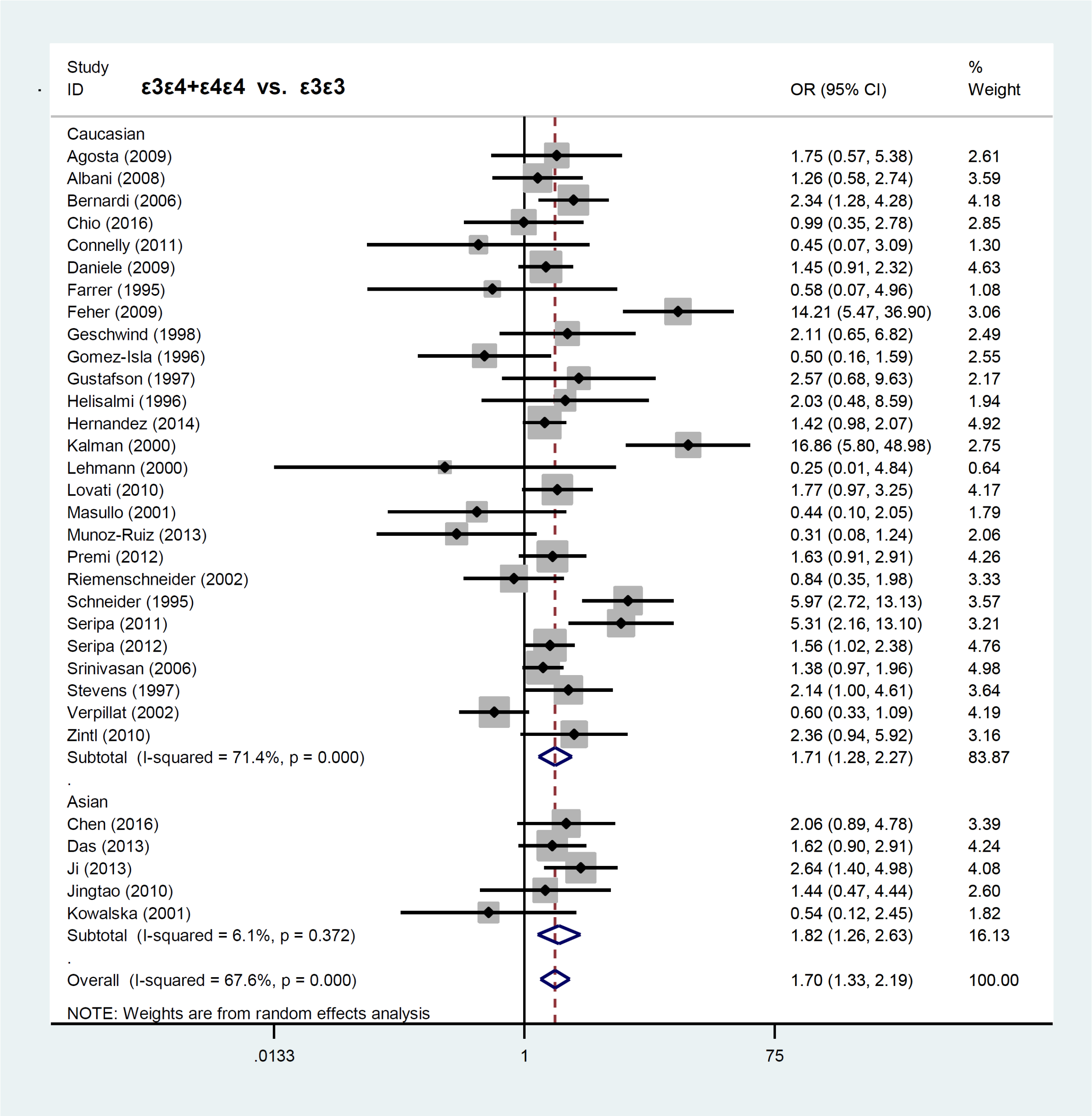

Supplementary Figure 7: Forest plot of subgroup analysis (Asian/Caucasian) of the ε3ε4+ε4ε4 vs. ε3ε3 model.

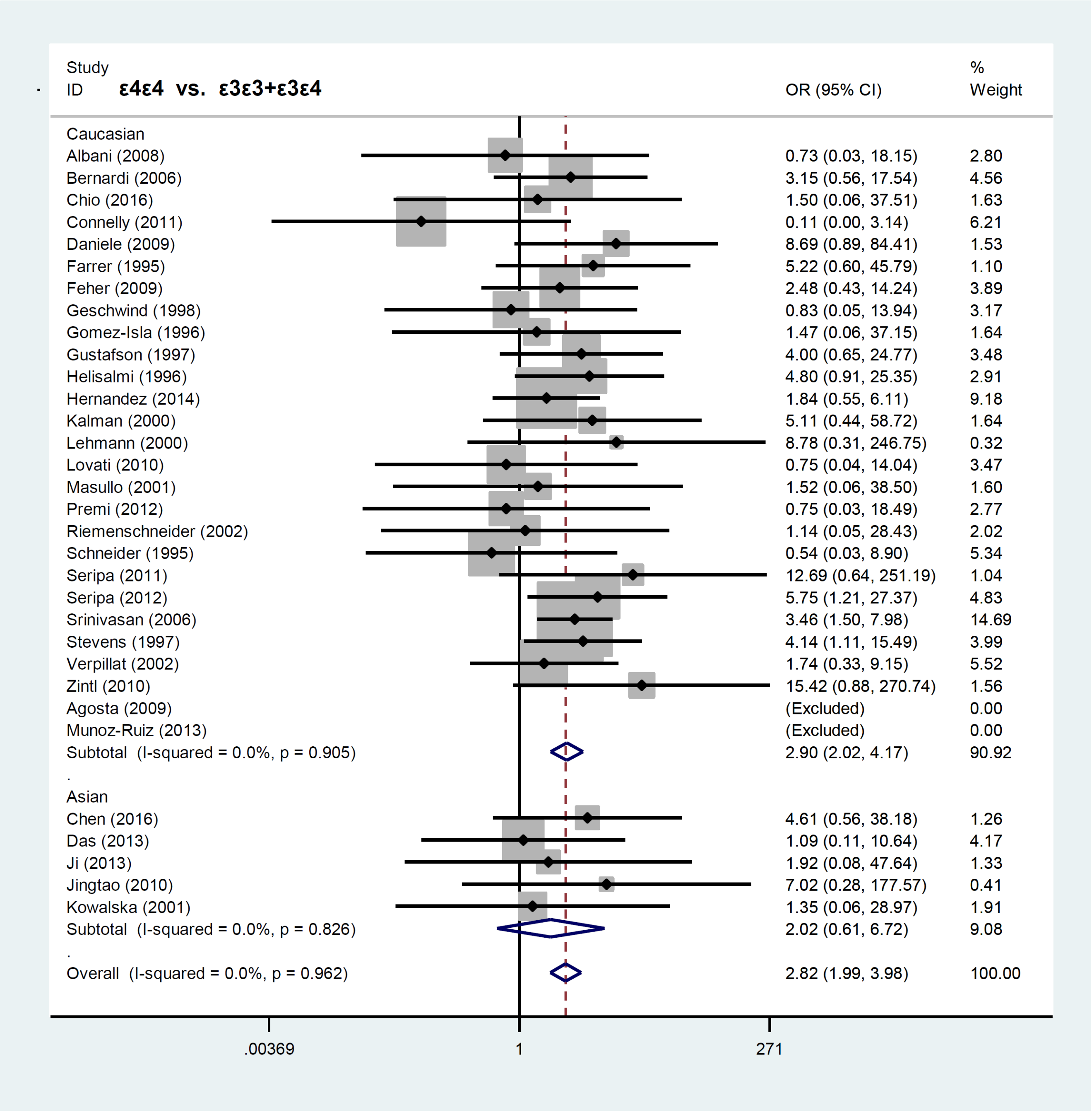

Supplementary Figure 8: Forest plot of subgroup analysis (Asian/Caucasian) of the ε4ε4 vs. ε3ε3+ε3ε4 model.

Supplementary Table 1: Characteristics of included studies. See Supplementary_Table_1

Supplementary Table 2: Subgroup analysis of association between *APOE* ε3/ε4/ε2 and FTLD risks for ε4 vs. ε2+ε3+ε4 carrier and ε2 vs. ε2+ε3+ε4 carrier models

	ε4 vs. ε2+ε3+ε4 carrier				ε2 vs. ε2+ε3+ε4 carrier			
**Subgroup**	**Study number**	**Sample size (case/control)**	**OR (95 % CI)**	***P***	**Study number**	**Sample size (case/control)**	**OR (95 % CI)**	***P***
**Ethnicity**								
**Caucasian**	42	3293/15547	1.49 (1.30–1.71)	**< 0.001**	27	1718/11092	0.99 (0.77–1.26)	0.922
**Asian**	5	218/2499	1.65 (1.19–2.29)	**0.003**	5	218/2499	0.62 (0.36–1.09)	0.097
Country								
**Italy**	14	1537/3118	1.53 (1.29–1.80)	**< 0.001**	9	839/2168	0.97 (0.70–1.34)	0.863
**China**	3	113/2030	1.92 (1.25–2.96)	**0.003**	3	113/2030	0.60 (0.29–1.26)	0.179
**USA**	5	178/3965	1.36 (0.80–2.32)	0.258	4	106/3394	1.12 (0.48–2.61)	0.797
**UK**	3	295/942	1.29 (0.79–2.11)	0.304	2	209/892	1.37 (0.11–17.09)	0.809
Source of control								
**PB**	46	3487/17846	1.51 (1.33–1.71)	**< 0.001**	31	1912/13391	0.95 (0.76–1.19)	0.640
**HB**	1	24/200	1.00 (0.28–3.56)	1.000	1	24/200	0.21 (0.03–1.58)	0.130
Clinical subtypes								
bvFTD	6	585/2510	1.53 (1.23–1.91)	**0.003**	4	373/2257	0.43 (0.21–0.87)	**0.019**
**SD**	4	94/1209	1.24 (0.75–2.03)	0.399	1	59/956	1.90 (0.19–18.97)	0.585
**PNFA**	3	81/403	1.87 (1.13–3.10)	**0.015**	2	66/200	0.40 (0.09–1.81)	0.237
**FTLD MND−**	2	96/442	0.85 (0.52–1.40)	0.528	1	23/114	0.21 (0.01–3.72)	0.289
**FTLD MND+**	3	104/1198	1.28 (0.86–1.90)	0.217	2	33/870	6.09 (2.41–15.40)	**< 0.001**
NOS								
**score > 6**	43	3375/16344	1.51 (1.32–1.73)	**< 0.001**	30	1831/13122	0.95 (0.75–1.20)	0.683
**Score < = 6**	4	136/1702	1.36 (0.88–2.10)	0.165	2	105/469	0.55 (0.16–1.98)	0.363

PB: population-based; HB: hospital-based; bvFTD: behavior variant frontotemporal dementia; SD: semantic dementia; PNFA: progressive non-fluent aphasia; FTLD: Frontotemporal lobar degeneration; MND: motor neuron disease; NOS: Newcastle-Ottawa scale; *P* value of association test less than 0.05 was shown in bold.

Supplementary Table 3: Subgroup analysis of association between *APOE* ε2 and FTLD risks for ε2 vs. ε3 and ε2 vs. ε2+ε3+ε4 allele models

	ε2 vs. ε3				ε2 vs. ε2+ε3+ε4 allele			
**Subgroup**	**Study number**	**Sample size (case/control)**	**OR (95 % CI)**	***P***	**Study number**	**Sample size (case/control)**	**OR (95 % CI)**	***P***
Ethnicity								
**Caucasian**	29	1854/11162	1.16 (0.91–1.48)	0.242	29	1854/11162	1.05 (0.85–1.31)	0.637
**Asian**	**5**	218/2499	0.67(0.33–1.38)	0.278	5	218/2499	0.67 (0.34–1.31)	0.239
Country								
**Italy**	10	839/2168	1.03 (0.73–1.46)	0.853	10	839/2168	0.98 (0.70–1.37)	0.889
**China**	3	113/2030	0.69 (0.29–1.62)	0.393	3	113/2030	0.66 (0.28–1.54)	0.333
**USA**	4	106/3394	1.22 (0.48–3.12)	0.671	4	106/3394	1.10 (0.44–2.75)	0.845
**UK**	4	345/962	1.74 (0.63–4.77)	0.282	4	345/962	1.58 (0.61–4.09)	0.347
Source of control								
**PB**	31	1912/13391	1.10 (0.87–1.39)	0.442	31	1912/13391	1.01 (0.81–1.25)	0.938
**HB**	3	160/270	0.87 (0.23–3.24)	0.832	3	160/270	0.87 (0.27–2.86)	0.822
Clinical subtypes								
**bvFTD**	4	373/2257	0.72 (0.49–1.06)	0.093	4	373/2257	0.69 (0.47–1.01)	0.057
**SD**	2	59/956	0.82 (0.35–1.92)	0.644	2	59/956	0.82 (0.35–1.92)	0.652
**PNFA**	1	60/200	2.29 (1.00–5.25)	0.051	1	0/1003	2.02 (0.89–4.61)	0.094
**FTLD MND−**	2	50/149	1.88 (0.78–4.57)	0.162	2	50/149	1.86 (0.77–4.51)	0.166
**FTLD MND+**	3	45/905	0.71 (0.27–1.91)	0.500	3	45/905	0.69 (0.26–1.83)	0.453
NOS								
**score > 6**	28	1800/11889	1.03 (0.82–1.31)	0.776	28	1800/11889	0.98 (0.78–1.22)	0.852
**Score < = 6**	6	272/1772	1.09 (0.87–1.37)	0.273	6	272/1772	1.23 (0.62–2.41)	0.556

PB: population-based; HB: hospital-based; bvFTD: behavior variant frontotemporal dementia; SD: semantic dementia; PNFA: progressive non-fluent aphasia; FTLD: Frontotemporal lobar degeneration; MND: motor neuron disease; NOS: Newcastle-Ottawa scale.

Supplementary Table 4: Subgroup analysis of association between *APOE* ε3/ε2 genotype frequency and FTLD risks for ε2ε2 vs. ε3ε3 and ε3ε2 vs. ε3ε3 models

	ε2ε2 vs. ε3ε3				ε3ε2 vs. ε3ε3			
**Subgroup**	**Study number**	**Sample size (case/control)**	**OR (95 % CI)**	***P***	**Study number**	**Sample size (case/control)**	**OR (95 % CI)**	***P***
Ethnicity								
**Caucasian**	17	784/7595	1.75 (0.95–3.22)	0.072	27	1186/8627	0.94 (0.78–1.13)	0.493
**Asian**	5	160/2113	1.73 (0.60–4.97)	0.311	5	160/2113	0.41 (0.20–0.83)	**0.013**
Country								
**Italy**	5	289/1008	0.84 (0.25–2.83)	0.773	10	603/1776	1.00 (0.76–1.31)	0.987
**China**	3	105/1735	3.41 (0.72–16.00)	0.121	3	81/1723	0.44 (0.17–1.10)	0.079
**USA**	4	65/2527	2.58 (0.56–11.83)	0.221	4	65/2527	1.21 (0.58–2.52)	0.609
**UK**	1	132/555	2.00 (0.18–22.31)	0.572	2	142/649	0.99 (0.62–1.58)	0.972
Source of control								
**PB**	21	923/9533	2.02 (1.16–3.50)	**0.013**	31	1325/10565	0.89 (0.74–1.07)	0.208
**HB**	1	21/175	0.28 (0.02–4.86)	0.379	1	21/175	0.11 (0.01–1.89)	0.129
HWE								
***P* > 0.05**	20	828/9337	1.85 (1.05–3.27)	**0.034**	30	1312/10543	0.88 (0.74–1.06)	0.182
***P* < 0.05**	1	21/175	0.28 (0.02–4.86)	0.379	1	21/175	0.11 (0.01–1.89)	0.129
Clinical subtypes								
**bvFTD**	3	112/1565	1.75 (0.35–8.85)	0.500	4	259/1798	0.73 (0.47–1.12)	0.149
**SD**	1	24/555	4.35 (0.20–93.49)	0.348	2	45/714	0.88 (0.36–2.15)	0.778
**PNFA**	-	-	–	-	1	44/159	1.82 (0.69–4.78)	0.227
**FTLD MND−**	-	-	–	-	1	20/94	1.48 (0.37–5.96)	0.579
**FTLD MND+**	1	18/555	5.40 (0.25–117.01)	0.283	2	21/649	0.58 (0.15–4.78)	0.429
NOS								
**score > 6**	18	845/8381	1.64 (0.90–3.00)	0.107	28	1247/9413	0.89 (0.74–1.06)	0.194
**Score < = 6**	4	99/1327	2.15 (0.72–6.41)	0.169	4	99/1327	0.65 (0.29–1.48)	0.307

PB: population-based; HB: hospital-based; HWE: Hardy-Weinberg Equilibrium; bvFTD: behavior variant frontotemporal dementia; SD: semantic dementia; PNFA: progressive non-fluent aphasia; FTLD: Frontotemporal lobar degeneration; MND: motor neuron disease; NOS: Newcastle-Ottawa scale; P value of association test less than 0.05 was shown in bold.

Supplementary Table 5: Subgroup analysis of association between *APOE* ε3/ε2 genotype frequency and FTLD risks for ε3ε2+ε2ε2 vs. ε3ε3 and ε2ε2 vs. ε3ε3+ε3ε4 models

	ε3ε2+ε2ε2 vs. ε3ε3				ε2ε2 vs. ε3ε3+ε3ε4			
**Subgroup**	**Study number**	**Sample size (case/control)**	**OR (95 % CI)**	***P***	**Study number**	**Sample size (case/control)**	**OR (95 % CI)**	***P***
Ethnicity								
**Caucasian**	27	1186/8627	1.04 (0.79–1.37)	0.772	17	784/7595	1.80 (0.98–3.32)	0.058
**Asian**	5	160/2113	0.44 (0.20–0.95)	**0.036**	5	160/2113	1.97 (0.68–5.69)	0.213
Country								
**Italy**	10	603/1776	0.93 (0.60–1.45)	0.761	5	289/1008	0.89 (0.26–3.03)	0.853
**China**	3	81/1723	0.37 (0.10–1.43)	0.148	3	105/1735	3.69 (0.78–17.34)	0.099
**USA**	4	65/2527	1.55 (0.75–3.20)	0.234	4	65/2527	2.62 (0.57–11.93)	0.213
**UK**	2	142/649	1.87 (0.29–12.21)	0.513	1	132/555	2.11 (0.19–23.45)	0.543
Source of control								
**PB**	31	1325/10565	0.96 (0.74–1.25)	0.758	21	923/9533	2.09 (1.20–3.62)	**0.009**
**HB**	1	21/175	0.15 (0.01–2.53)	0.188	1	21/175	0.33 (0.02–5.85)	0.452
HWE								
***P* > 0.05**	30	1312/10543	0.95 (0.72–1.25)	0.716	20	828/9337	1.93 (0.88–8.88)	**0.024**
***P* < 0.05**	1	21/175	0.15 (0.01–2.53)	0.188	1	21/175	0.33 (0.88–8.88)	0.452
Clinical subtypes								
**bvFTD**	4	259/1798	0.77 (0.50–1.18)	0.225	3	112/1565	1.90 (0.37–9.64)	0.440
**SD**	2	45/714	0.87 (0.36–2.13)	0.762	1	24/555	4.52 (0.21–96.68)	0.335
**PNFA**	1	44/159	1.82 (0.69–4.78)	0.227	-	-	–	-
**FTLD MND−**	1	20/94	1.48 (0.37–5.96)	0.579	-	-	–	-
**FTLD MND+**	2	21/649	0.59 (0.16–2.25)	0.442	1	18/555	5.98 (0.28–129.10)	0.254
NOS								
**score > 6**	28	1247/9413	0.95 (0.71–1.26)	0.703	18	845/8381	1.71 (0.94–3.13)	0.082
**Score < = 6**	4	99/1327	0.95 (0.36–2.47)	0.910	4	99/1327	2.39 (0.79–7.26)	0.124

PB: population-based; HB: hospital-based; HWE: Hardy-Weinberg Equilibrium; bvFTD: behavior variant frontotemporal dementia; SD: semantic dementia; PNFA: progressive non-fluent aphasia; FTLD: Frontotemporal lobar degeneration; MND: motor neuron disease; NOS: Newcastle-Ottawa scale; *P* value of association test less than 0.05 was shown in bold.

Supplementary Table 6: Publication bias analysis

Comparison	Begg's test (continuity corrected)		Egger's test	
	Z	*P*	T	*P*
ε4 vs ε3 allele	1.30	0.192	−0.43	0.668
ε4 vs ε2 allele	0.71	0.477	−0.38	0.705
ε4 vs ε2+ε3+ε4 allele	1.27	0.204	−0.41	0.683
ε4 vs ε2+ε3+ε4 carrier	0.55	0.582	0.18	0.855
ε4ε4 vs ε3ε3	0.96	0.335	−1.33	0.194
ε4ε4 vs ε3ε3+ε3ε4	1.07	0.284	−1.57	0.127
ε3ε4 vs ε3ε3	0.83	0.408	−0.06	0.953
ε3ε4+ε4ε4 vs ε3ε3	0.66	0.506	0.01	0.992
ε2 vs ε3 allele	0.44	0.657	0.01	0.995
ε2 vs ε2+ε3+ε4 allele	0.56	0.573	−0.20	0.842
ε2 vs ε2+ε3+ε4 allele	0.56	0.573	−0.20	0.842
ε2 vsε2+ε3+ε4 carrier	0.66	0.506	−0.11	0.911
ε2ε2 vs ε3ε3	0.23	0.822	−0.34	0.735
ε3ε2 vs ε3ε3	0.34	0.733	−0.01	0.992
ε3ε2+ε2ε2 vs ε3ε3	0.05	0.961	0.35	0.730
ε2ε2 vs ε3ε3+ε3ε2	0.39	0.693	−0.45	0.655

Supplementary Table 7: The search strategy of databases for meta-analysis. See Supplementary_Table_7

Supplementary Table 8: PRISMA 2009 Checklist. See Supplementary_Table_8
