# Supplementary material for: Updated meta-analysis of the role of APOE ε2/ε3/ε4 alleles in frontotemporal lobar degeneration
Source: Oncotarget. 2017 Apr 21;8(27):43721–32. doi: 10.18632/oncotarget.17341 (PMC5546436; doi:10.18632/oncotarget.17341)
Supplement: Supplementary file 2 [file oncotarget-08-43721-s002.docx]

Supplementary Table S1: Characteristics of included studies

|  |  |  |  |  | Genotype | | | | | |  |  |  |  |  |
| --- | --- | --- | --- | --- | --- | --- | --- | --- | --- | --- | --- | --- | --- | --- | --- |
| First author | **Year** | **Country** | **Ethnicity** | **Group** | **ε3ε3** | **ε3ε2** | **ε2ε2** | **ε3ε4** | **ε4ε4** | **ε2ε4** | **Total** | **Clinical subtypes** | **Source of control** | **Genotyping assay** | **NOS score** |
| Agosta | 2009 | Italy | Caucasian | case | 20 | 3 | 0 | 8 | 0 | 0 | 31 | bvFTD |  | gene sequencing | 8 |
|  |  |  |  | control | 35 | 10 | 1 | 8 | 0 | 2 | 56 |  | PB |  |  |
| Albani | 2008 | Italy | Caucasian | case | 49 | 9 | 1 | 12 | 0 | 2 | 73 | FTLD |  | PCR-RFLP | 8 |
|  |  |  |  | control | 113 | 14 | 1 | 21 | 1 | 1 | 151 |  | PB |  |  |
| Bagnoli | 2013 | Italy | Caucasian | case | - | - | - | - | - | 20^&^ | 156 | FTLD |  | PCR-RFLP | 9 |
|  |  |  |  | control | - | - | - | - | - | 20^&^ | 272 |  | PB |  |  |
| Balasa | 2014 | Spain | Caucasian | case | - | - | - | - | - | 5^&^ | 25 | FTLD |  | PCR-RFLP | 8 |
|  |  |  |  | control | - | - | - | - | - | 9^&^ | 37 |  | PB |  |  |
| Bernardi | 2006 | Italy | Caucasian | case | 61 | 4 | 1 | 28 | 4 | 2 | 100 | FTLD |  | PCR-RFLP | 8 |
|  |  |  |  | control | 116 | 31 | 4 | 24 | 2 | 3 | 180 |  | PB |  |  |
| Boccardi | 2003 | Italy | Caucasian | case | - | - | - | - | - | 3^#^ | 18 | FTLD |  | NA | 8 |
|  |  |  |  | control | - | - | - | - | - | 5^#^ | 50 |  | PB |  |  |
| Borroni | 2005 | UK | Caucasian | case | - | - | - | - | - | 18^&^ | 51 | bvFTD |  | PCR-RFLP | 8 |
|  |  |  |  | case | - | - | - | - | - | 10^&^ | 27 | SD |  |  |  |
|  |  |  |  | case | - | - | - | - | - | 4^&^ | 8 | PNFA |  |  |  |
|  |  |  |  | control | - | - | - | - | - | 10^&^ | 50 |  | PB |  |  |
| Chen | 2016 | China | Asian | case | 19 | 1 | 0 | 7 | 1 | 0 | 28 | bvFTD |  | TaqMan assay | 7 |
|  |  |  |  | case | 36 | 6 | 1 | 8 | 1 | 2 | 54 | SD |  |  |  |
|  |  |  |  | control | 802 | 154 | 8 | 156 | 8 | 21 | 1149 |  | PB |  |  |
| Chio | 2016 | Italy | Caucasian | case | 37 | 8 | 0 | 5 | 0 | 2 | 52 | FTLD+ALS |  | gene sequencing | 8 |
|  |  |  |  | control | 169 | 24 | 0 | 22 | 1 | 7 | 223 |  | PB |  |  |
| Connelly | 2011 | England | Caucasian |  | 11 | 2 | 1 | 5 | 0 | 0 | 19 | FTLD |  | NA | 7 |
|  |  |  |  | control | 3 | 1 | 1 | 2 | 1 | 1 | 9 |  | PB |  |  |
| Daniele | 2009 | Italy | Caucasian | case | 70 | 13 | 0 | 35 | 3 | 6 | 127 | FTLD |  | NA | 7 |
|  |  |  |  | control | 222 | 33 | 1 | 82 | 1 | 4 | 343 |  | PB |  |  |
| Das | 2013 | India | Asian | case | 53 | 3 | 2 | 21 | 1 | 1 | 81 | FTLD |  | PCR-RFLP | 6 |
|  |  |  |  | control | 195 | 19 | 1 | 47 | 3 | 4 | 269 |  | PB |  |  |
| Fabre | 2001 | Sweden | Caucasian | case | - | - | - | - | - | 33^&^ | 64 | FTLD |  | microsequencing | 8 |
|  |  |  |  | control | - | - | - | - | - | 10^&^ | 47 |  | PB |  |  |
| Farrer | 1995 | USA | Caucasian | case | 5 | 2 | 0 | 0 | 1 | 2 | 10 | FTLD |  | PCR-RFLP | 6 |
|  |  |  |  | control | 765 | 143 | 7 | 227 | 38 | 24 | 1204 |  | PB |  |  |
| Feher | 2009 | Hungary | Caucasian | case | 7 | 2 | 0 | 20 | 2 | 7 | 38 | FTLD |  | PCR-RFLP | 8 |
|  |  |  |  | control | 113 | 24 | 2 | 21 | 4 | 0 | 164 |  | PB |  |  |
| Galimberti | 2009 | Italy | Caucasian | case | - | - | - | - | - | 47^&^ | 161 | bvFTD |  | PCR-RFLP | 9 |
|  |  |  |  | case | - | - | - | - | - | 3^&^ | 7 | PNFA |  |  |  |
|  |  |  |  | case | - | - | - | - | - | 2^&^ | 8 | SD |  |  |  |
|  |  |  |  | case | - | - | - | - | - | 56^&^ | 212 | FTLD |  |  |  |
|  |  |  |  | control | - | - | - | - | - | 28^&^ | 203 |  | PB |  |  |
| Geschwind | 1998 | USA | Caucasian | case | 18 | 2 | 0 | 11 | 1 | 1 | 33 | FTLD |  | PCR-RFLP | 7 |
|  |  |  |  | control | 19 | 4 | 1 | 5 | 1 | 0 | 30 |  | PB |  |  |
| Gomez-Isla | 1996 | USA | Caucasian | case | 21 | 5 | 1 | 4 | 0 | 0 | 31 | FTLD |  | PCR-RFLP | 7 |
|  |  |  |  | control | 82 | 14 | 0 | 30 | 1 | 2 | 129 |  | PB |  |  |
| Gustafson | 1997 | Sweden | Caucasian | case | 10 | 1 | 2 | 3 | 4 | 1 | 21 | FTLD |  | PCR-RFLP | 6 |
|  |  |  |  | control | 22 | 0 | 0 | 4 | 2 | 1 | 29 |  | PB |  |  |
| Helisalmi | 1996 | Finland | Caucasian | case | 5 | 0 | 0 | 1 | 3 | 0 | 9 | FTLD |  | PCR-RFLP | 7 |
|  |  |  |  | control | 38 | 6 | 0 | 10 | 5 | 1 | 60 |  | PB |  |  |
| Hernandez | 2014 | Spain | Caucasian | case | 114 | 9 | 1 | 37 | 3 | 4 | 168 | FTLD |  | real-time PCR | 7 |
|  |  |  |  | control | 2178 | 317 | 17 | 508 | 29 | 34 | 3083 |  | PB |  |  |
| Ingelson | 2001 | Sweden | Caucasian | case | - | - | - | - | - | 17^&^ | 33 | FTLD |  | microsequencing | 7 |
|  |  |  |  | control | - | - | - | - | - | 7^&^ | 39 |  | PB |  |  |
| Ji | 2013 | China | Asian | case | 40 | 2 | 0 | 18 | 0 | 2 | 62 | FTLD |  | PCR-RFLP | 8 |
|  |  |  |  | control | 288 | 37 | 1 | 48 | 1 | 6 | 381 |  | PB |  |  |
| Jingtao | 2010 | China | Asian | case | 16 | 2 | 1 | 4 | 0 | 0 | 23 | FTLD |  | PCR-RFLP | 8 |
|  |  |  |  | control | 368 | 61 | 4 | 63 | 1 | 3 | 500 |  | PB |  |  |
| Kalman | 2000 | Hungary | Caucasian | case | 7 | 1 | 0 | 20 | 2 | 6 | 36 | FTLD |  | PCR | 8 |
|  |  |  |  | control | 59 | 9 | 0 | 10 | 1 | 0 | 79 |  | PB |  |  |
| Kowalska | 2001 | Japan | Asian | case | 21 | 0 | 0 | 2 | 0 | 1 | 24 | FTLD |  | PCR-SSCP and DNA sequencing | 5 |
|  |  |  |  | control | 136 | 28 | 11 | 22 | 2 | 1 | 200 |  | HB |  |  |
| Lehmann | 2000 | UK | Caucasian | case | 4 | 6 | 0 | 0 | 0 | 1 | 11 | FTLD |  | one-stage PCR | 8 |
|  |  |  |  | control | 83 | 11 | 0 | 35 | 1 | 6 | 136 |  | PB |  |  |
| Lovati | 2010 | Italy | Caucasian | case | 48 | 10 | 0 | 17 | 0 | 0 | 75 | FTLD |  | PCR-RFLP | 7 |
|  |  |  |  | control | 370 | 54 | 3 | 70 | 4 | 5 | 506 |  | PB |  |  |
| Masullo | 2001 | Italy | Caucasian | case | 17 | 3 | 0 | 2 | 0 | 1 | 23 | FTLD MND- |  | PCR-RFLP | 7 |
|  |  |  |  | case | 3 | 0 | 0 | 0 | 0 | 0 | 3 | FTLD MND+ |  |  |  |
|  |  |  |  | control | 84 | 10 | 0 | 18 | 1 | 1 | 114 |  | PB |  |  |
| Minthon | 1997 | Sweden | Caucasian | case | - | - | - | - | - | 7^&^ | 25 | FTLD |  | IEF, PCR and reverse DNA hybridization | 7 |
|  |  |  |  | control | - | - | - | - | - | 7^&^ | 26 |  | PB |  |  |
|  |  |  |  | case | - | - | - | - | - | 7^#^ | 50 | FTLD |  |  |  |
|  |  |  |  | control | - | - | - | - | - | 7^#^ | 52 |  | PB |  |  |
| Morenas-Rodriguez | 2016 | Spain | Caucasian | case | - | - | - | - | - | 11^&^ | 32 | FTLD |  | NA | 7 |
|  |  |  |  | control | - | - | - | - | - | 18^&^ | 74 |  | PB |  |  |
| Munoz-Ruiz | 2013 | Finland | Caucasian | case | 14 | 2 | 0 | 4 | 0 | 0 | 20 | FTLD |  | PCR-RFLP | 7 |
|  |  |  |  | control | 12 | 1 | 0 | 11 | 0 | 1 | 25 |  | PB |  |  |
| Pickering-Brown | 1995 | UK | Caucasian | case | - | - | - | 6* | 43* | 5* | 54* | FTLD MND- |  | NA | 5 |
|  |  |  |  | case | - | - | - | 1* | 18* | 5* | 24* | FTLD MND+ |  |  |  |
|  |  |  |  | case | - | - | - | 8* | 72* | 16* | 96* | FTLD |  | NA |  |
|  |  |  |  | control | - | - | - | 4* | 56* | 10* | 70* |  | HB |  |  |
| Pickering-Brown | 2000 | UK | Caucasian | case | - | - | - | 14* | 128* | 34* | 176* | FTLD |  | PCR-RFLP | 5 |
|  |  |  |  | control | - | - | - | 4* | 56* | 10* | 70* |  | HB |  |  |
| Premi | 2012 | Italy | Caucasian | case | 37 | 7 | 0 | 19 | 0 | 3 | 66 | PNFA |  | PCR-RFLP | 8 |
|  |  |  |  | case | 19 | 2 | 0 | 7 | 0 | 0 | 28 | SD |  |  |  |
|  |  |  |  | control | 144 | 15 | 0 | 40 | 1 | 0 | 200 |  | PB |  |  |
| Riemenschneider | 2002 | Germany | Caucasian | case | 35 | 6 | 1 | 8 | 0 | 2 | 52 | FTLD |  | PCR-RFLP | 7 |
|  |  |  |  | control | 117 | 28 | 2 | 31 | 1 | 3 | 182 |  | PB |  |  |
| Rosso | 2002 | Netherlands | Caucasian | case | - | - | - | - | - | 37^&^ | 98 | FTLD |  | PCR-RFLP | 7 |
|  |  |  |  | control | - | - | - | - | - | 159^&^ | 561 |  | PB |  |  |
|  |  |  |  | case | - | - | - | - | - | 43^#^ | 196 | FTLD |  |  |  |
|  |  |  |  | control | - | - | - | - | - | 172^#^ | 1122 |  | PB |  |  |
| Ruiz | 2014 | Spain | Caucasian | case | - | - | - | - | - | 64^&^ | 261 | FTLD |  | NA | 7 |
|  |  |  |  | control | - | - | - | - | - | 257^&^ | 1596 |  | PB |  |  |
| Schneider | 1995 | USA | Caucasian | case | 9 | 2 | 0 | 21 | 0 | 0 | 32 | FTLD |  | PCR-RFLP | 7 |
|  |  |  |  | control | 1249 | 233 | 10 | 437 | 51 | 51 | 2031 |  | PB |  |  |
| Seripa | 2011 | Italy | Caucasian | case | 29 | 5 | 0 | 16 | 3 | 1 | 54 | FTLD |  | PCR-RFLP | 7 |
|  |  |  |  | control | 73 | 16 | 0 | 9 | 0 | 1 | 99 |  | PB |  |  |
| Seripa | 2012 | Italy | Caucasian | case | 131 | 16 | 0 | 50 | 8 | 2 | 207 | bvFTD |  | Taqman assay | 7 |
|  |  |  |  | control | 204 | 29 | 0 | 56 | 2 | 5 | 296 |  | PB |  |  |
| Short | 2002 | USA | Caucasian | case | - | - | - | - | - | 26^#^ | 126 | FTLD |  | PCR-RFLP | 7 |
|  |  |  |  | case | - | - | - | - | - | 8^#^ | 54 | bvFTD |  |  |  |
|  |  |  |  | case | - | - | - | - | - | 3^#^ | 24 | PNFA |  |  |  |
|  |  |  |  | control | - | - | - | - | - | 75^#^ | 676 |  | PB |  |  |
| Sleegers | 2004 | Netherlands | Caucasian | case | - | - | - | - | - | 4^&^ | 6 | FTLD |  | PCR-RFLP | 7 |
|  |  |  |  | case | - | - | - | - | - | 39^&^ | 120 |  | PB |  |  |
|  |  |  |  | case | - | - | - | - | - | 4^#^ | 12 | FTLD |  |  |  |
|  |  |  |  | control | - | - | - | - | - | 42^#^ | 240 |  | PB |  |  |
| Srinivasan | 2006 | UK | Caucasian | case | 57 | 11 | 1 | 32 | 5 | 1 | 107 | bvFTD |  | PCR-RFLP | 7 |
|  |  |  |  | case | 20 | 4 | 0 | 5 | 2 | 0 | 31 | SD |  |  |  |
|  |  |  |  | case | 16 | 2 | 0 | 10 | 1 | 1 | 30 | FTLD MND+ |  |  |  |
|  |  |  |  | case | 111 | 20 | 1 | 53 | 11 | 2 | 198 | FTLD |  |  |  |
|  |  |  |  | control | 445 | 108 | 2 | 174 | 12 | 15 | 756 |  | PB |  |  |
| Steenland | 2010 | USA | Caucasian | case | - | - | - | - | - | 28^&^ | 72 | FTLD |  | NA | 7 |
|  |  |  |  | control | - | - | - | - | - | 203^&^ | 571 |  | PB |  |  |
| Stevens | 1997 | Netherlands | Caucasian | case | 14 | 6 | 0 | 11 | 3 | 0 | 34 | FTLD |  | PCR-RFLP | 8 |
|  |  |  |  | control | 315 | 80 | 7 | 134 | 13 | 12 | 561 |  | PB |  |  |
| van | 2014 | mixed | Caucasian | case | - | - | - | - | - | 21^&^ | 73 | FTLD MND- |  | NA | 7 |
|  |  |  |  | case | - | - | - | - | - | 28^&^ | 71 | FTLD MND+ |  |  |  |
|  |  |  |  | control | - | - | - | - | - | 108^&^ | 328 |  | PB |  |  |
|  |  |  |  | case | - | - | - | - | - | 23^#^ | 146 | FTLD MND- |  | NA |  |
|  |  |  |  | case | - | - | - | - | - | 31^#^ | 142 | FTLD MND+ |  |  |  |
|  |  |  |  | control | - | - | - | - | - | 120^#^ | 656 |  | PB |  |  |
| Venturelli | 2008 | Italy | Caucasian | case | - | - | - | - | - | 21^&^ | 71 | FTLD |  | PCR-RFLP | 8 |
|  |  |  |  | control | - | - | - | - | - | 22^&^ | 190 |  | PB |  |  |
| Verpillat | 2002 | France | Caucasian | case | 60 | 13 | 3 | 14 | 2 | 2 | 94 | FTLD |  | PCR-RFLP | 8 |
|  |  |  |  | control | 226 | 51 | 1 | 96 | 5 | 13 | 392 |  | PB |  |  |
| Villa | 2012 | Italy | Caucasian | case | - | - | - | - | - | 60^&^ | 290 | FTLD |  | PCR-RFLP | 7 |
|  |  |  |  | control | - | - | - | - | - | 63^&^ | 341 |  | PB |  |  |
| Zintl | 2010 | Germany | Caucasian | case | 42 | 7 | 0 | 14 | 10 | 0 | 73 | FTLD |  | LightCycler real-time PCR | 7 |
|  |  |  |  | control | 33 | 12 | 0 | 8 | 0 | 1 | 54 |  | PB |  |  |

&, carrier data; #, allele data; *, the allele of ε2, ε3, ε4 and ε2+ε3+ε4; PB: population-based; HB: hospital-based; bvFTD: behavior variant frontotemporal dementia; FTLD: Frontotemporal lobar degeneration; PNFA: progressive non-fluent aphasia; SD: semantic dementia; ALS: amyotrophic lateral sclerosis; MND: motor neuron disease; PCR-RFLP: polymerase chain reaction-restriction fragment length polymorphism; NA: not available; IEF, isoelectric focusing; PCR: polymerase chain reaction; PCR-SSCP: polymerase chain reaction-single strand conformation polymorphism.
