# Supplementary material for: Updated meta-analysis of the role of APOE ε2/ε3/ε4 alleles in frontotemporal lobar degeneration
Source: Oncotarget. 2017 Apr 21;8(27):43721–32. doi: 10.18632/oncotarget.17341 (PMC5546436; doi:10.18632/oncotarget.17341)
Supplement: Supplementary file 3 [file oncotarget-08-43721-s003.docx]

Supplementary Table S7: The search strategy of databases for meta-analysis

**Database 1^st^: PubMed**

| #1 "Frontotemporal Lobar Degeneration"[Mesh] Sort by: PublicationDate  #2 ((((((Degeneration, Frontotemporal Lobar) OR Degenerations, Frontotemporal Lobar) OR Frontotemporal Lobar Degenerations) OR Lobar Degeneration, Frontotemporal) OR Lobar Degenerations, Frontotemporal) OR FTLD) OR FTLDs Sort by: PublicationDate  #3 (#1 OR #2)  #4 "Apolipoproteins E"[Mesh] Sort by: PublicationDate  #5 ((((((((ApoE) OR Apo E) OR Apo-E) OR Apolipoprotein E Isoproteins) OR Isoproteins, Apolipoprotein E) OR Apo E Isoproteins) OR Isoproteins, Apo E) OR Apoproteins E) OR Apoprotein (E) Schema: all Sort by: PublicationDate  #6 (#4 OR #5)  #7 (#3 AND #6) |
| --- |

**Database 2^nd^:EMBASE**

| #1 'frontotemporal dementia'/exp  #2 'dementia, frontotemporal'  #3 'frontal dementia'  #4 'frontal lobe dementia'  #5 'frontotemporal dementias'  #6 'frontotemporal lobar degeneration'  #7 'ftd (frontotemporal dementia)'  #8 'ftld'  #9 'pick complex'  #10 (#1 OR #2 OR #3 OR #4 OR #5 OR #6 OR #7 OR #8 OR #9)  #11 'apolipoprotein e'/exp  #12 'apo e'  #13 'apolipoprotein e'  #14 'apoprotein e'  #15 (#11 OR #12 OR #13 OR #14)  #16 (#10 AND #15) |
| --- |

**Database 3^rd^: CENTRAL**

| #1 MeSH descriptor: [Frontotemporal Lobar Degeneration] explode all trees  #2 MeSH descriptor: [Apolipoproteins E] explode all trees  #3 Lobar Degenerations, Frontotemporal or Degenerations, Frontotemporal Lobar or Frontotemporal Lobar Degenerations or Lobar Degeneration, Frontotemporal or Lobar Degenerations, Frontotemporal (Word variations have been searched)  #4 Apo E or Apo-E or Apolipoprotein E Isoproteins (Word variations have been searched)  #5 (#1 or #3)  #6 (#2 or #4)  #7 (#5 and #6) |
| --- |

**Database 4^th^: Web of Science (WOS)**

| #1 TOPIC: (Frontotemporal Lobar Degeneration) OR TOPIC: (Degeneration, Frontotemporal Lobar) OR TOPIC: (Degenerations, Frontotemporal Lobar) OR TOPIC:(Frontotemporal Lobar Degenerations) OR TOPIC: (Lobar Degenerations, Frontotemporal) OR TOPIC: (FTLD) OR TOPIC: (FTLDs)  Timespan=All years  Search language=Auto  #2 TOPIC: (Apolipoproteins E) OR TOPIC: (ApoE) OR TOPIC: (Apo E) OR TOPIC: (Apo-E) OR TOPIC: (Apolipoprotein E Isoproteins) OR TOPIC: (Isoproteins, Apolipoprotein E) OR TOPIC: (Apo E Isoproteins) OR TOPIC: (Isoproteins, Apo E) OR TOPIC: (Apoproteins E) OR TOPIC: (Apoprotein (E))  Timespan=All years  Search language=Auto  #3 （#2 AND #1）  Timespan=All years  Search language=Auto |
| --- |
